# Research on subway settlement prediction based on the WTD-PSR combination and GSM-SVR model

**DOI:** 10.1038/s41598-025-02673-w

**Published:** 2025-05-26

**Authors:** Miren Rong, Chao Feng, Yinping Pang, Hailong Wang, Ying Yuan, Wensong Zhang, Lanxin Luo

**Affiliations:** 1 School of Urban Geology and Engineering, Hebei GEO University, Shijiazhuang, 050031 China; 2 Hebei Technology Innovation Center for Intelligent Development and Control of Underground Built Environment, Shijiazhuang, 050031 China; 3 Shijiazhuang College of Applied Technology, Shijiazhuang, 050800 China; 4 Shijiazhuang Tiedao University, Shijiazhuang, 050043 China

**Keywords:** Settlement prediction, Wavelet denoising, Phase space reconstruction, Support vector regression, Grid search method, Natural hazards, Computer science

## Abstract

Due to the speeding up of urban development and the rapid population expansion in China, the subway has become the preferred mode of transportation for people, and urban underground spaces are continuously being improved. However, during the construction of subways, surface settlement around the area is inevitably caused, which can significantly impact the safety of the building procedure and surrounding buildings. Therefore, accurately predicting surface settlement around subway tunnels is of great practical significance. This study proposes a GSM-SVR model for subway settlement prediction, based on WTD-PSR data preprocessing. The data of surface settlement data from three measurement points in a section of the 1st Line of the urban rail transit system was taken as the research subject. By upgrading the one-dimensional settlement data sequence to a multi-dimensional data sequence, and utilizing the Grid Search Method Optimized Support Vector Regression (GSM-SVR) regression model to predict subway settlement with small sample data, the aim is to offer a more precise and reliable data analysis method and theoretical approach for small sample subway settlement prediction. First, wavelet denoising (WTD) is applied to the field measured data, and the denoised time series data is subjected to phase space reconstruction (PSR) to obtain multi-dimensional time series data. The validity of the embedding dimension determined by the phase space reconstruction is verified, providing rich multi-dimensional features for the subsequent prediction models. Based on the reconstructed data, traditional Support Vector Regression (SVR) models and SVR models optimized by the Grid Search Method (GSM) are constructed. Furthermore, Particle Swarm Optimization (PSO), Gray Wolf Optimization (GWO), Marine Predators Algorithm (MPA), and Whale Optimization Algorithm (WOA) are introduced to optimize the SVR model, and the prediction performance is compared with that of the Long Short-Term Memory (LSTM) model. By comparing the prediction accuracy of the seven models, the results show that the Grid Search Method optimized SVR model performs the best in the light of prediction accuracy, with a Mean Absolute Percentage Error (MAPE) of 2.96%, a Mean Absolute Error (MAE) of 0.028 mm, a Root Mean Square Error (RMSE) of 0.032 mm, and a coefficient of determination (R^2^) of 0.995. Compared with the other six models, the three-error metrics are reduced by 25.25%–64.72%, 32.93%–68.81%, and 34.43%–70.53%, respectively, and the R^2^ value is increased by 0.75%–6.39%, significantly outperforming traditional empirical models. These results indicate that the GSM-SVR model based on WTD-PSR significantly outperforms single-algorithm optimization strategies, making it more suitable for predicting future ground settlement data. This approach provides a reusable hybrid framework for small-sample settlement prediction in subway systems, offering improved guidance for practical engineering applications.

## Introduction

Recently, with the speeding up of urbanization and the increasing concentration of urban populations, the load on cities has intensified, leading to a rapid development period for subway construction in China. Due to the high efficiency and strong transportation capacity of subways^1^, which serve as a green and efficient mode of transportation, they play an essential role in alleviating urban pressure, facilitating urban mobility, and promoting urban development. During subway construction, surface settlement is a critical factor affecting the safety of the project, and its impact on the safety of major infrastructure such as subways will persist over the long term. Specifically, urban safety was threatened by the settlement caused by subway construction^2^. For example, the settlement mentioned above can lead to the formation of voids beneath the embankment, leading to surface subsidence or collapse^3^. Excessive differential settlement of existing underground pipelines may cause water leakage and gas leakage, leading to accidents^4,5^. If early warning measures are not promptly, the sort of settlement can result in unrecoverable accidents and pose a serious risk to public safety and property^6^. Meanwhile, during subway construction, it is crucial to quickly and accurately obtain surface settlement data to guarantee the safety of the building project. Therefore, it is of paramount importance to accurately predict the surface deformations caused by underground construction for safety.

During different stages of underground engineering construction, the monitoring data changes accordingly, indicating that the data variations are influenced by changes in working conditions, and thus belong to a one-dimensional time series. The data variation curve reflects the progress of the underground construction, providing a better basis for ensuring the safety of subsequent construction stages. With the development of big data technologies, numerous predictive modeling methods have emerged. Currently, methods such as numerical simulation, artificial neural networks, and support vector machines are widely used to predict surface deformations caused by underground construction. Among these, support vector machines (SVM) are made a widely used method in recent years for data prediction due to their simplicity, strong data mining capability, powerful nonlinear fitting ability, and accurate prediction results. However, single-algorithm approaches fail to meet the accuracy requirements for data prediction. Due to noise interference during subway construction, the prediction results exhibit randomness, as they overlook the effective integration of data preprocessing and predictive modeling. In contrast, the SVM regression model, after wavelet denoising and the elimination of outliers, utilizes phase space reconstruction to obtain multidimensional data, which better adapts to the model for more accurate data prediction.

The structure of the following sections in this study is as follows: Section “Literature review” summarizes the project research related to ground settlement observations. Section “Methods for processing settlement deformation data and prediction models” provides a discussion of the methodology, describing the preprocessing of data and the regression prediction model. Section “Case analysis” outlines the engineering background, data processing, and regression prediction of the data, analyzing the changes in prediction accuracy before and after model optimization. Section “Conclusion” presents the conclusion.

## Literature review

During the construction of metro tunnels, timely predictions of surface settlement trends are a crucial component of informatized construction. Over the past few decades, many researchers have developed new prediction models and methods to make it more accurate and efficient in surface settlement prediction, trying to benefit from machine learning^7^ and achieving a series of results. Many methods have already been implemented in the prediction of surface settlement, including Random Forest^8,9^, XGBoost^10,11^, Extreme Learning Machine^12,13^, Radial Basis Function Neural Networks^14^, and Backpropagation Neural Networks^15,16^. Other methods include the application of regression models^17^, which input multiple parameters for regression on settlement data. Due to their powerful nonlinear fitting ability, these models can easily recognize the linear trends of surface settlement data, resulting in predictions of surface settlement, such as Support Vector Machine Regression^18^. Some researchers have also employed other machine learning methods and compared the prediction results of the models used^19,20^. Mahmoodzadeh, et al.^21^ employed seven intelligent methods, including Long Short-Term Memory (LSTM), Deep Neural Networks (DNN), K-Nearest Neighbors (KNN), Gaussian Process Regression (GPR), Support Vector Regression (SVR), Decision Trees (DT), and Linear Regression (LR), to study the maximum surface settlement resulting from urban tunnels. With the continuous emergence of new prediction models, it has become apparent that a single model cannot meet practical applications. In the calculation process, the inherent characteristics of the data are often overlooked, which may lead to inaccurate prediction results^22^. To further enhance the precision of surface settlement predictions, some researchers have introduced hybrid models. Elbaz, et al.^23^proposed an improved Particle Swarm Optimization (PSO) algorithm, which was combined with the Adaptive Neuro-Fuzzy Inference System (ANFIS) based on Fuzzy C-Means (FCM) clustering for predicting the performance of metro tunnel shield machines. Hu, et al.^24^ proposed a hybrid prediction model based on an improved particle swarm optimization (IPSO) algorithm and a BP neural network to predict the surface settlement caused by the construction of rectangular pipe-jacking tunnels. The model’s prediction accuracy and stability were validated through a real engineering case. Moghaddasi and Noorian-Bidgoli^25^ introduced an optimized Artificial Neural Network hybrid model, which uses the Imperial Competitive Algorithm (ICA-ANN) and can accurately predict the largest surface deformation caused by structural damage and environmental factors. Su, et al.^26^ proposed a hybrid prediction model based on an S-shaped growth curve, with superior performance compared to individual fitting curves. Due to the existence of noise in the raw data, some researchers^27^ selected the Kalman filtering method as a denoising technique and used Radial Basis Function Neural Networks and Exponential Smoothing algorithms to predict the processed surface settlement data. The combination of Support Vector Machines (SVMs) with other optimization methods can also improve model accuracy. Zhang, et al.^28^ proposed a hybrid algorithm that integrates PSO and Least Squares Support Vector Machine (LSSVM) to simulate the system’s reaction to tunnel damage caused by construction. Shults, et al.^29^ utilized machine learning techniques, including regression analysis and neural network regression, to predict the spatial displacement of interchanges caused by ground settlement during metro construction, finding that the optimized neural network regression provided the most accurate prediction model for geotechnical monitoring in urban environments. Liu, et al.^30^ proposed an intelligent prediction method based on the deep forest model for predicting strata settlement in subsea metal mining. This method effectively improved prediction accuracy and provided a scientific basis for ensuring mining safety. It is a great success for machine learning in settlement prediction^31^, and a good complement to traditional settlement prediction methods^32^.

Additionally, some scholars have directly applied settlement data for prediction, eliminating the need for soil parameters. Examples of such approaches include deep learning models, such as Long Short-Term Memory (LSTM) networks^33,34^, and regression models, such as the Autoregressive Moving Average (ARMA) model^35^. Among these methods, deep learning networks, particularly LSTM models, have attracted considerable attention due to their remarkable capability to handle sophisticated patterns and non-linear correlations. Ye, et al.^36^ utilized field monitoring data from Fiber Bragg Grating (FBG) sensors and employed a Time-Series Backpropagation Neural Network (TS-BPNN) model to estimate surface settlement resulting from shield tunnel construction. Their study validated the effectiveness of this method during both rapid and slow settlement phases. Hong, et al.^37^ developed an LSTM-based model for time-series prediction of consolidation settlement on soft soil foundations. The model was compared with 120 different training data scenarios and traditional methods (e.g., hyperbolic method and Asaoka method), showing that the LSTM model outperforms conventional methods in light of prediction accuracy. Regression models, on the other hand, are widely used owing to their capability to detect trends and patterns in past data, they can also be used for time-series prediction. The core of time-series prediction lies in using historical data values to forecast future values. By combining regression models with time-series data prediction, the relationships between historical data points can be analyzed to predict future values. Liu, et al.^38^ combined Differential Evolution (DE) and Gray Wolf Optimization (GWO) algorithms to optimize the parameters of a Support Vector Regression (SVR) model, successfully predicting settlement during the construction of a high embankment, validating the efficacy of this integrated method in improving the accuracy of embankment settlement prediction. Mozaffari, et al.^39^ developed a hybrid model combining Support Vector Regression (SVR) and Particle Swarm Optimization (PSO) for groundwater level time-series prediction. They emphasized that this model effectively captures the dynamic characteristics of groundwater level variations and optimizes the SVR parameters to enhance prediction precision. Zhang, et al.^40^ proposed a hybrid model that combines joint denoising techniques with an enhanced Gray Wolf Optimized SVR (EGWO-n-SVR) model, specifically targeting the preprocessing and parameter optimization of high embankment settlement time-series data to improve the accuracy and robustness of regression models in predicting high embankment settlement. These studies indicate that the use of SVR regression models for time-series prediction yields good results, and combining them with other optimization methods further enhances prediction accuracy. Considering that SVR’s performance is significantly determined by its hyperparameters, traditional optimization algorithms (such as Particle Swarm Optimization) may often get trapped in local minima during the iteration process^41^. In regression prediction, multiple features are typically used as input sequences to improve prediction accuracy. Given that settlement data is inherently time-series data, it needs to be transformed into a suitable form for simulation prediction before regression. The key lies in constructing lagged features. By using data from previous time points as input features, the prediction of settlement values involves the application of phase space reconstruction. However, studies combining phase space reconstruction with regression models for surface settlement prediction are relatively scarce, especially those integrating phase space reconstruction with Support Vector Machine Regression. Phase space reconstruction overcomes the limitation of traditional SVR models, which can only handle static feature data, by transforming time-series data (a typical dynamic system) into a format suitable for regression analysis, allowing SVR to effectively learn and predict. An SVR model is employed for surface settlement prediction, optimized using five different algorithms, and an LSTM network is introduced for comprehensive comparison. The results show that the SVR model optimized using grid search yields the best performance improvement.

The contributions of this study are as follows:

(1) This study focuses on resolving noise errors in the one-dimensional characteristics of subway surface settlement monitoring data. By comparing the matching properties of different wavelet functions (db, Haar, Symlet) with the monitoring signal, the optimal wavelet function is selected. Furthermore, a comparison is made between wavelet denoising and Fast Fourier Transform (FFT): FFT relies on spectral analysis to set a cutoff frequency for filtering specific frequency band noise, while the wavelet method achieves multi-scale separation of signal and noise through adaptive selection of the basis function. The experiments demonstrate that the wavelet method is more advantageous in suppressing non-stationary noise and retaining local features, avoiding the issue of signal detail loss caused by global frequency domain truncation in FFT.

(2) To address the lack of temporal correlation in one-dimensional data, the phase space reconstruction technique is employed to construct a multi-dimensional system matrix. The delay time is determined using the C–C method, and the embedding dimension is further confirmed using the GP algorithm. The results of the embedding dimension selection are validated, showing promising performance. Based on the daily monitoring characteristics of subway settlement, the original sequence is transformed into a sample set of"features from the previous N days → settlement value on the N + 1 day"through reconstruction. This method not only validates the rationality of the embedding dimension selection but also directly establishes an explicit mapping between historical data and future settlement values, providing structured input features for the support vector machine regression model, which is suitable for time-series prediction tasks.

(3) The hyperparameters of the Support Vector Machine Regression (SVR) model were optimized using Grid Search Method (GSM), significantly improving the model’s prediction accuracy and generalization capability. Further comparative analysis reveals that, compared to five other optimization algorithms and Long Short-Term Memory (LSTM) networks, the proposed GSM-SVR model based on the WTD-PSR combination not only provides prediction results that better align with the actual settlement trend under small sample conditions, but also demonstrates stronger stability and adaptability in data fluctuation scenarios. This model offers a more scientific and practical theoretical tool for dynamic early warning and risk management of subway construction settlement.

## Methods for processing settlement deformation data and prediction models

Settlement deformation is inevitable, and the direct or indirect losses it causes are substantial. Monitoring, analyzing, and predicting settlement and deformation are critical tasks for its prevention and control. Therefore, a Support Vector Regression (SVR) model is employed, with prior wavelet denoising and phase space reconstruction applied to the raw data. The process of denoising and the principles of reconstruction are explained, as well as the structure of the prediction model.

### Wavelet denoising

In the monitoring of settlement data, the accuracy and reliability of the data are the cornerstone for ensuring the validity of conclusions, as well as for rational decision-making. It is important to acknowledge that any monitoring process is inevitably influenced by various factors, and the observed settlement time-series data will inevitably be contaminated by substantial noise, which significantly affects the predictive accuracy of models. Complex noise factors such as temperature, humidity, and pressure can cause a certain degree of distortion in the data collected by sensors^42^. Therefore, it is necessary to effectively identify, quantify, and eliminate these errors in order to obtain high-quality surface settlement data that closely approximates the true situation.

Donoho and Johnstone^43^ proposed a threshold denoising method, namely the Wavelet Threshold Denoising (WTD) method, which is based on wavelet transformation. In the field of signal processing, wavelet denoising has significant advantages over traditional denoising techniques, as it effectively separates the signal from the noise. In recent years, it has become widely used as a mathematical tool for denoising, compression, and encoding of one-dimensional and two-dimensional signals^44^. The WTD method has excellent time–frequency localization properties, and by utilizing its multi-scale and multi-resolution characteristics, it can remove noise while preserving the details of the signal, thereby achieving better denoising results^45^. When a noisy signal is input, the wavelet transform decomposes it into low-frequency coefficients (signal) and high-frequency coefficients (noise). The low-frequency coefficients (signal) can be further decomposed into multiple layers until the ideal wavelet coefficients are obtained. The process is divided into three main steps: (1) decompose the noisy signal into several layers of wavelet coefficients using the wavelet transform; (2) apply a threshold function to process each layer of wavelet coefficients and remove noise-related components; (3) reconstruct the signal using the inverse wavelet transform.

Denoising principle of wavelet transform:

Surface settlement data can be considered as a time-varying signal. Let the surface settlement observation data be denoted as *f*(*t*), which can be expressed as the sum of the true surface settlement signal *x*(*t*) and the noise interference signal *s*(*t*). That is,

1 $$f\left(t\right)=x\left(t\right)+s\left(t\right)$$

**Fig. 1:**
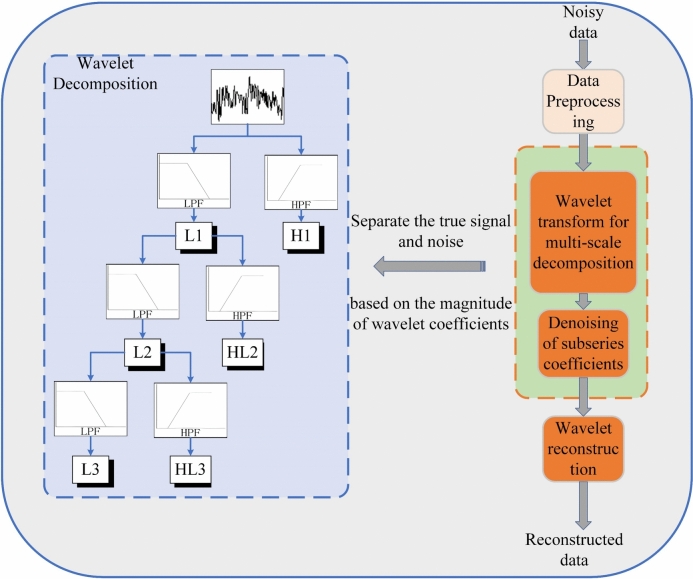
Principle of wavelet denoising.

The essence of wavelet transformation is to retain the effective part of the monitoring data and separate the obtained monitoring time series into *x*(*t*) (the true signal) and *s*(*t*) (the noise signal), thus effectively removing the noise from the observed signal. In general, under a multi-scale decomposition scheme, the wavelet coefficients of the true surface settlement signal *x*(*t*) and the noise signal *s*(*t*) exhibit different characteristics. Typically, the wavelet coefficients of *x*(*t*) have larger magnitudes and show relatively stable increases with scale, while the wavelet coefficients of *s*(*t*) have smaller magnitudes and their increase gradually diminishes to zero as the scale increases. Therefore, by setting an appropriate threshold, the smaller wavelet coefficients, which are primarily attributed to noise, are removed, while the larger coefficients are retained. The signal is then reconstructed using the inverse wavelet transform, effectively eliminating the noise from the signal. Figure 1 illustrates the principle of wavelet denoising.

The commonly used threshold functions are the hard threshold function and the soft threshold function. Let *W*_*i,k*_ denotes the wavelet coefficients of the original surface settlement data *f*(*t*), and the wavelet coefficients after thresholding are denoted as ${\widehat{W}}_{i,k}$.

Hard threshold:

2 $${\widehat{W}}_{i,k}=\left\{\begin{array}{c}{W}_{i,k}, \left|{\omega }_{i,k}\right|\ge \lambda \\ 0, \left|{\omega }_{i,k}\right|<\lambda \end{array}\right.$$

Soft threshold:

3 $${\widehat{W}}_{i,k}=\left\{\begin{array}{c}sign\left({W}_{i,k}\right)\left(\left|{\omega }_{i,k}\right|-\lambda \right), \left|{\omega }_{i,k}\right|\ge \lambda \\ 0, \left|{\omega }_{i,k}\right|<\lambda \end{array}\right.$$

where: *λ* is the threshold, and *sign*(*W*_*i,k*_) is the sign function. The threshold *λ* can be computed using the universal thresholding method. That is:

4 $$\lambda =\sigma \sqrt{2\text{ln}N}$$

where *σ* is the estimated standard deviation of the noise, which can be estimated from the first-level detail coefficients of the wavelet decomposition of the original surface settlement data, and *N* is the data length. After determining the threshold and processing the wavelet coefficients, the denoised surface settlement data $\widehat{f}\left(t\right)$ is reconstructed using the Inverse Discrete Wavelet Transform (IDWT):

5 $$\widehat{f}\left(t\right)=\sum_{j}\sum_{k}{\widehat{W}}_{i,k}{\varphi }_{j,k}\left(t\right)$$

Denoising performance evaluation: The effectiveness of the wavelet transform denoising (WTD) is assessed by comparing the characteristics of the surface settlement data before and after denoising, such as data variance, signal-to-noise ratio (SNR), and other metrics. The SNR calculation formula is:

6 $$SNR=10{\text{log}}_{10}\left(\frac{{\sum }_{t}{x}^{2}\left(t\right)}{{\sum }_{t}{\left(x\left(t\right)-\widehat{x}\left(t\right)\right)}^{2}}\right)$$

where *x*(*t*) is the true signal component in the original data, and $\widehat{x}\left(t\right)$ is the estimated true signal component in the denoised data. A higher SNR indicates better denoising performance, meaning the denoised data is closer to the true surface settlement signal.

### Phase space reconstruction

#### Principle of phase space reconstruction

Phase space reconstruction is a key step in the chaotic analysis and processing of time series data and is widely used in time series data analysis^46^. In surface settlement studies, the collected monitoring data is typically in the form of a one-dimensional time series and often exhibits chaotic characteristics, thus requiring phase space reconstruction of the original data. According to Takens’embedding theorem^47^, a multi-dimensional time series matrix can be reconstructed from a one-dimensional chaotic time series. If the embedding dimension and time delay *τ* are chosen appropriately, the reconstructed phase space will be equivalent to the original system and will have the same topological structure. The analysis and prediction of chaotic time series are performed within this reconstructed space. In this study, based on phase space reconstruction theory, the monitoring data are mapped into a high-dimensional space^48^.

Surface settlement monitoring data exists in the form of a time series *x*(*n*), where *n* denotes the sampling index in the time series. Phase space reconstruction is applied to the one-dimensional settlement monitoring data series *x*(*n*) to obtain a multi-dimensional time series matrix.

Let the reconstructed multi-dimensional time series be denoted as ${\left\{{X}_{i}\right\}}_{i=1}^{N}$, expressed as follows:

7 $$\left\{\begin{array}{c}{X}_{1}=\left[x\left(1\right),x\left(1+\tau \right),\cdots \text{x}\left(1+\left(\text{m}-1\right)\uptau \right)\right]\\ {X}_{2}=\left[x\left(2\right),x\left(2+\tau \right),\cdots \text{x}\left(2+\left(\text{m}-1\right)\uptau \right)\right]\\ \vdots \\ {X}_{N}=\left[x\left(N\right),x\left(N+\tau \right),\cdots \text{x}\left(\text{N}+\left(\text{m}-1\right)\uptau \right)\right]\end{array}\right.$$

In the equation, *m* represents the embedding dimension, which determines the number of elements included in each phase point in the reconstructed phase space, which represents the dimensionality of the spatial expansion of the one-dimensional data. *τ* is the delay parameter, which defines the time interval between adjacent elements when selecting the phase point elements. An appropriate value of *τ* allows the preservation of the dynamic characteristics of the original data in the reconstructed phase space. The number of phase points in the reconstructed phase space is *M* = *N-*(*m-1*)*τ*. Where *N* is the number of data points in the original surface settlement time series *x*(n). The determination of *M* is closely related to the length of the original data, the embedding dimension, and the delay parameter.

#### The simultaneous calculation of the delay parameter and embedding dimension

In chaotic time series phase space reconstruction, the selection of the delay parameter *τ* and embedding dimension *m* is crucial for describing the characteristic changes in the original time-delay data series^49^. The determination of the delay time and embedding dimension forms the basis of phase space reconstruction^50^. Traditional views consider *m* and *τ* to be independent and require separate or sequential determination. Recent studies, however, have shown that *m* and *τ* are closely related, and a relationship between the two parameters can be established through the delay time window *τ*_*w*_ = (*m-1*)*τ*.

The C–C algorithm^51^ uses the correlation integral to construct a statistical measure, which simultaneously computes *τ* and *τ*_*w*_, and consequently determines the embedding dimension. The correlation integral is defined as follows:

8 $$C\left(m,N,r,\tau \right)=\frac{2}{M\left(M-1\right)}\sum_{1\ll i<j<M}H\left(r-{d}_{ij}\right)$$

For surface settlement data, *M* represents the number of phase points; *m* is the embedding dimension; *N* is the number of data points in the surface settlement time series, which corresponds to the length of the collected settlement data; *r* is the neighborhood radius, used to define the similarity between two points in the phase space; *d*_*ij*_ = $\Vert {X}_{i}-{X}_{j}\Vert$, is the Euclidean distance between two points *X*_*i*_ and *X*_*j*_ in the phase space. The difference between phase points can be measured by calculating the Euclidean distance; *H*(*z*) is the Heaviside step function, which is defined as follows:

9 $$H\left(z\right)=\left\{\begin{array}{c}1, z>0\\ 0,z\ll 0\end{array}\right.$$

Define the test statistics as follows:

10 $${S}_{1}\left(m,\text{N},\text{r},\uptau \right)=C\left(m,\text{N},\text{r},\uptau \right)-{C}^{m}\left(1,\text{N},\text{r},\uptau \right)$$

In the above calculation, the block averaging method is used. That is:

11 $${S}_{2}\left(m,\text{N},\text{r},\uptau \right)=\frac{1}{\tau }\sum_{s=1}^{\tau }\left[{C}_{s}\left(m,\frac{N}{\tau },r,\tau \right)-{C}_{s}^{m}\left(1,\frac{N}{\tau },r,\tau \right)\right]$$

Define the differential as follows:

12 $$\Delta S\left(m,\text{N},\text{r},\uptau \right)=max\left\{S\left(m,\text{N},\text{r},\uptau \right)\right\}-min\left\{S\left(m,\text{N},\text{r},\uptau \right)\right\}$$

For the surface settlement data, let *N* = 1000, *m* = 2,3,4,5, *r* = *k σ*/2, *k* = 1,2,3,4, *σ* is the standard deviation of the surface settlement time series *x*(*n*). Let *τ* = 1,2, …,200, and the mean values of *S*_*2*_(*m, N, r, τ*) and *S*_*1*_(*m, N, r, τ*), and denoted as $\overline{{S }_{2}}\left(\tau \right), \overline{{S }_{1}}\left(\tau \right)$, and the differences $\Delta \overline{{S }_{2}}\left(\tau \right)$ and $\Delta \overline{{S }_{1}}\left(\tau \right)$ are computed. The first local minimum point of $\Delta \overline{{S }_{1}}\left(\tau \right)$ is selected as the delay parameter *τ*.

13 $$\overline{{S }_{2}}\left(\tau \right)=\frac{1}{16}\sum_{m=2}^{5}\sum_{k=1}^{4}{S}_{2}\left(m,\text{N},\text{r},\uptau \right)$$

14 $$\Delta \overline{{S }_{2}}\left(\tau \right)=\frac{1}{4}\sum_{m=2}^{5}\Delta {S}_{2}$$

The periodic points of $\left|\overline{{S }_{1}}\left(\tau \right)-\overline{{S }_{2}}\left(\tau \right)\right|$ are selected as the optimal embedding window *τ*_*w*_, where $\left|\overline{{S }_{1}}\left(\tau \right)-\overline{{S }_{2}}\left(\tau \right)\right|$ exhibits significant local peaks at the periodic points, which better reflect the intrinsic structural characteristics of the surface settlement data, thereby making the embedding window *τ*_*w*_ more reliable. Consequently, the embedding dimension is:

15 $$m=\frac{{\tau }_{w}}{\tau }+1$$

By closely integrating the various formulas of this method with the surface settlement data, the delay parameter *τ*, embedding window *τ*_*w*_ and embedding dimension *m* are determined. This enables the reconstructed phase space of the surface settlement data to more accurately reflect the characteristics and intrinsic patterns of the original data, providing a high-quality data foundation for further studies such as chaos analysis and prediction model development.

### Support vector machine regression mode

Support Vector Machine (SVM) is a machine learning method first proposed by Vapnik^52^. It is typically used for three main objectives: classification, regression, and pattern recognition. The SVM used for classification is referred to as the Support Vector Classifier (SVC), while the SVM used for regression is called the Support Vector Regression (SVR)^53^.Similar to a multilayer perceptron, SVM is used for classification and prediction. Dou, et al.^54^ transformed the constraints in SVM, further extending the method to Support Vector Regression (SVR). It was found that SVR can address both classification and linear or nonlinear regression problems, while also enabling the construction of linear regression functions in a new high-dimensional feature space, thereby reducing model complexity^55^. The surface settlement monitoring data can be represented as a series of sample points (*x*_*i*_*,y*_*i*_)(*x*_*i*_ ∈ *R*^*n*^ represents the input variables related to surface settlement, *y*_*i*_ ∈ *R*^*n*^ represents the corresponding observed surface settlement values, and *i* = 1,2,…,l, with l denoting the number of samples). The core idea is to map the low-dimensional input space (formed by *x*_*i*_) to a high-dimensional feature space using a nonlinear mapping *φ*(*x*), and then construct the optimal linear regression function in the feature space.

16 $$y=f\left(x\right)=\omega \varphi \left(x\right)+b$$

In the equation, *φ*(*x*) is a nonlinear mapping function that transforms the input variables of the original surface settlement data into a higher-dimensional space more suitable for linear regression analysis. *ω* is the weight vector, which determines the importance of each feature in the regression function, while *b* is the bias term, which adjusts the position of the regression hyperplane.

According to the structural risk minimization principle, the regression problem can be solved by minimizing the structural risk *R*_*str*_ to determine the parameters *ω* and *b*.

17 $$\text{minRstr}=\frac{1}{2}\left(\omega \cdot \omega \right)+\frac{c}{l}\sum_{i=1}^{l}\left|f\left({x}_{i}\right)-{y}_{i}\right|$$

In the equation, *c* represents the penalty factor, and *ε* is the insensitive loss coefficient, which is defined as follows:

18 $${\left|f\left({x}_{i}\right)-{y}_{i}\right|}_{\varepsilon }=\left\{\begin{array}{c}\left|f\left({x}_{i}\right)-{y}_{i}\right|-\varepsilon , \left|f\left({x}_{i}\right)-{y}_{i}\right|\ge \varepsilon \\ 0, \left|f\left({x}_{i}\right)-{y}_{i}\right|<\varepsilon \end{array}\right.$$

Its significance lies in determining an error tolerance range, within which errors are not included in the loss function. This helps to enhance the robustness of the model. For surface settlement data, due to factors such as measurement errors, setting a reasonable *ε* can prevent the model from being overly sensitive to small errors.

By introducing slack variables *ξ*_i_ and *ξ*_i_^*^, the above expression can be transformed into an equivalent optimization problem, namely:

19 $$min\frac{1}{2}\Vert {\omega }^{2}\Vert +c\sum_{i=1}^{l}\left({\xi }_{i}+{\xi }_{i}^{*}\right)$$

20 $$s.t.\left\{\begin{array}{c}\left(\left(\omega \cdot \varphi \left({x}_{i}\right)\right)+b\right)-{y}_{i}\le \varepsilon +{\xi }_{i}\\ {y}_{i}-\left(\left(\omega \cdot \varphi \left({x}_{i}\right)\right)+b\right)\le \varepsilon +{\xi }_{i}^{*}\\ {\xi }_{i}\ge 0,{\xi }_{i}^{*}\ge 0 i=\text{1,2},\cdots ,l\end{array}\right.$$

Further introducing Lagrange multipliers *ɑ*_*i*_ and *ɑ*_i_^*^, the above optimization problem is transformed into its dual form.

21 $$max\sum_{i=1}^{l}{y}_{i}\left({\alpha }_{i}^{*}-{\alpha }_{i}\right)-\varepsilon \sum_{i=1}^{l}\left({\alpha }_{i}^{*}+{\alpha }_{i}\right)-\frac{1}{2}\sum_{i,j=1}^{l}\left({\alpha }_{i}^{*}-{\alpha }_{i}\right)\left({\alpha }_{j}^{*}-{\alpha }_{j}\right)k\left({x}_{i},{x}_{j}\right)$$

22 $$s.t.\left\{\begin{array}{c}\sum_{i=1}^{l}\left({\alpha }_{i}^{*}-{\alpha }_{i}\right)\\ 0\le {\alpha }_{i}\le c 0\le {\alpha }_{i}^{*}\le c i=\text{1,2},\cdots ,l\end{array}\right.$$

In the equation, *k(x*_*i*_, *x*_*j*_) = *φ(x*_*i*_*)·φ(x*_*j*_*)* represents the kernel function.

Commonly used kernel functions include the radial basis function (RBF), linear kernel, and polynomial kernel, among others. The most widely applied kernel is the radial basis function, which exhibits strong learning capability. It is applicable in various scenarios, including low-dimensional, high-dimensional, small-sample, and large-sample cases, and possesses a broad convergence domain. In this study, the radial basis function kernel is selected.

### Subway settlement prediction method based on the WTD-PSR combined with GSM-SVR

**Fig. 2:**
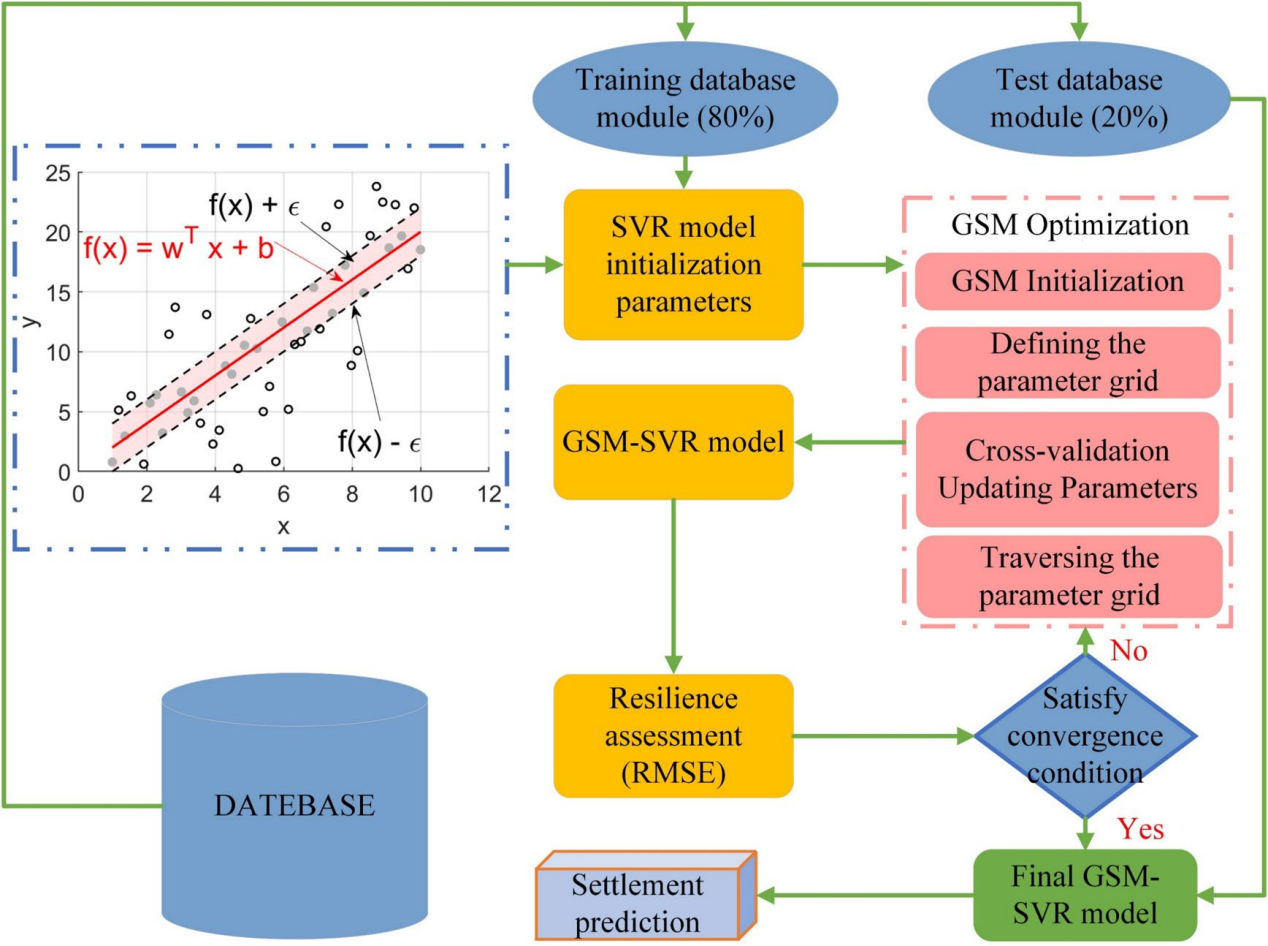
Flowchart of model construction selection.

Subway settlement monitoring data represents a typical time series with complex nonlinear characteristics, exhibiting chaotic variations in temporal patterns. To address these challenges, we employ the phase space reconstruction methodology outlined in Sect. “Phase Space Reconstruction”. This reconstruction mechanism effectively transforms the original settlement measurements into multidimensional phase space trajectories. This study validates the reliability of the phase space reconstruction method through numerical simulations and predictive analyses conducted on the MATLAB computational platform. The performance of the model is influenced by the values of its hyperparameters^56^. The model in this study requires optimization of fewer hyperparameters. Compared with other hyperparameter optimization algorithms, the Grid Search Method (GSM) is simple to use and highly operational. Therefore, the GSM algorithm is employed to optimize the parameters of the traditional Support Vector Regression (SVR) model, thereby establishing a time series prediction model for the settlement sequence. The flowchart of this study is shown in Fig. 2, and the main steps are as follows:

1) Sample data collection: The settlement data in this study came from the monitoring data of the Trimble DINI03 electronic level instrument, with a data collection frequency of once per day. To ensure the accuracy of the data, all sensors were calibrated before installation, and their measurement results were verified using the calibration data.

2) Data processing: The collected settlement data underwent wavelet threshold denoising to obtain a denoised data sequence. The denoised sequence was then subjected to phase space reconstruction, determining the embedding dimension and delay time.

3) Traditional SVR model prediction: The entire dataset was divided into training and testing sets based on a certain ratio and in chronological order.

4) Grid Search Method for Hyperparameter Optimization: Initial parameters for the grid search method were set, specifically the hyperparameters to be optimized for the SVR model. The SVR model was trained using the training set, and the accuracy of different parameter combinations was compared to identifying the optimal parameter combination (c, g) using the grid search method.

5) Performance testing of the optimized SVR model: The performance of the SVR model under optimal hyperparameters was tested using the test set data.

## Case analysis

### Project overview

This project is located in a certain city and concerns the section of the No. 1 Line of the urban rail transit system. The important buildings and structures above the Yingzi Section include the Luchao Gou Railway Bridge and the Beijing Road Turn Bridge (South and North). The railway bridge is a Class I risk source, while the highway bridges are all Class II risk sources, with a vertical crossing relationship to the tunnel section below. The tunnel section beneath the railway bridge and highway turn bridges is constructed using the “blind excavation” method, with a standard horseshoe-shaped, single-hole, single-track cross-section. According to the excavation and survey tests, the main strata in the area consist of Quaternary Upper Pleistocene rounded gravel and pebbles deposited by alluvial and fluvial riverbeds, with mixed fill soil widely distributed at the surface.

**Fig. 3:**
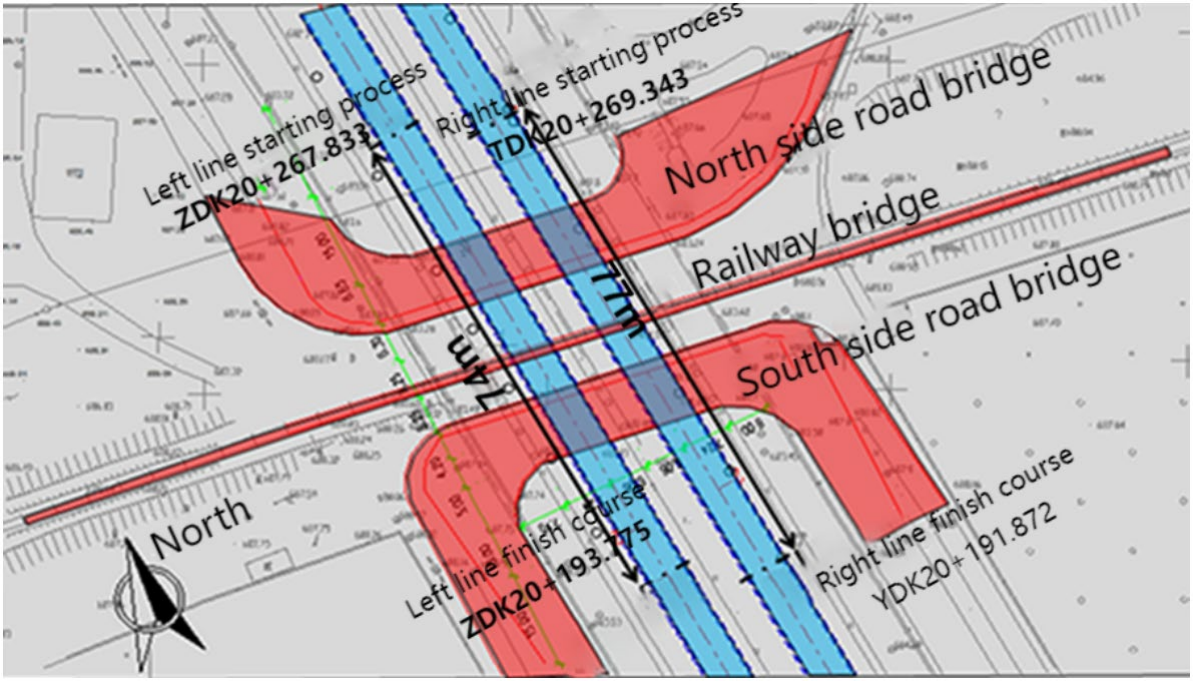
Plan view of the spatial relationship between the section of Yingzi, crossing beneath the railway and highway bridges.

**Fig. 4:**
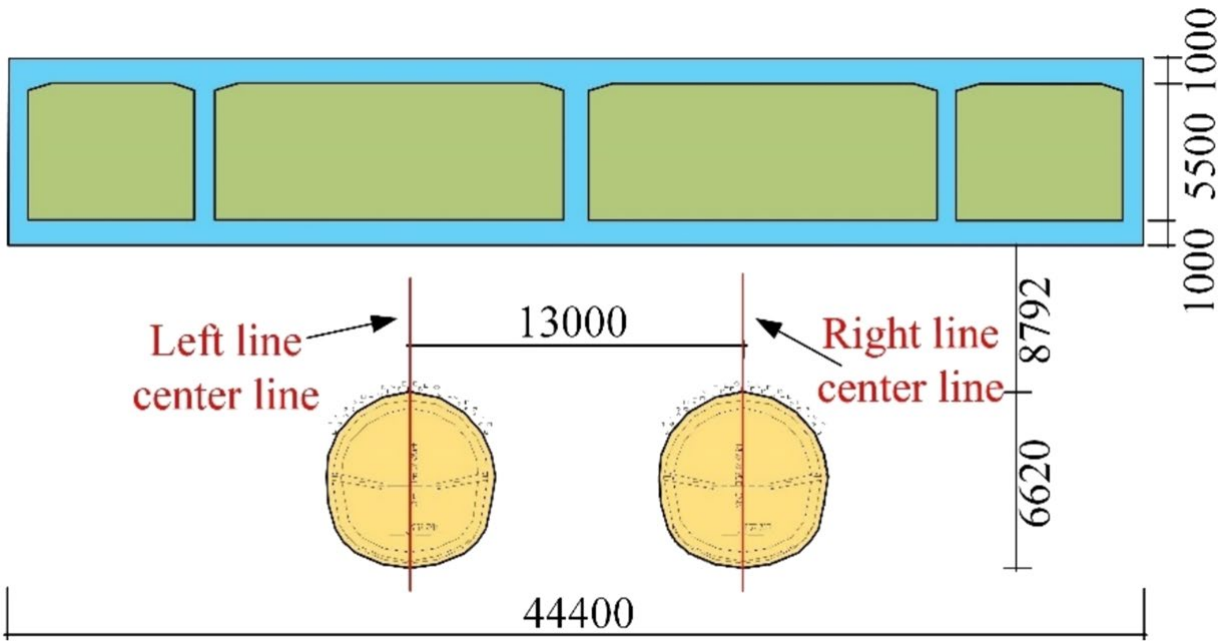
Schematic diagram showing the vertical alignment of the section tunnel underpass the Lu Caogou Bridge.

**Fig. 5:**
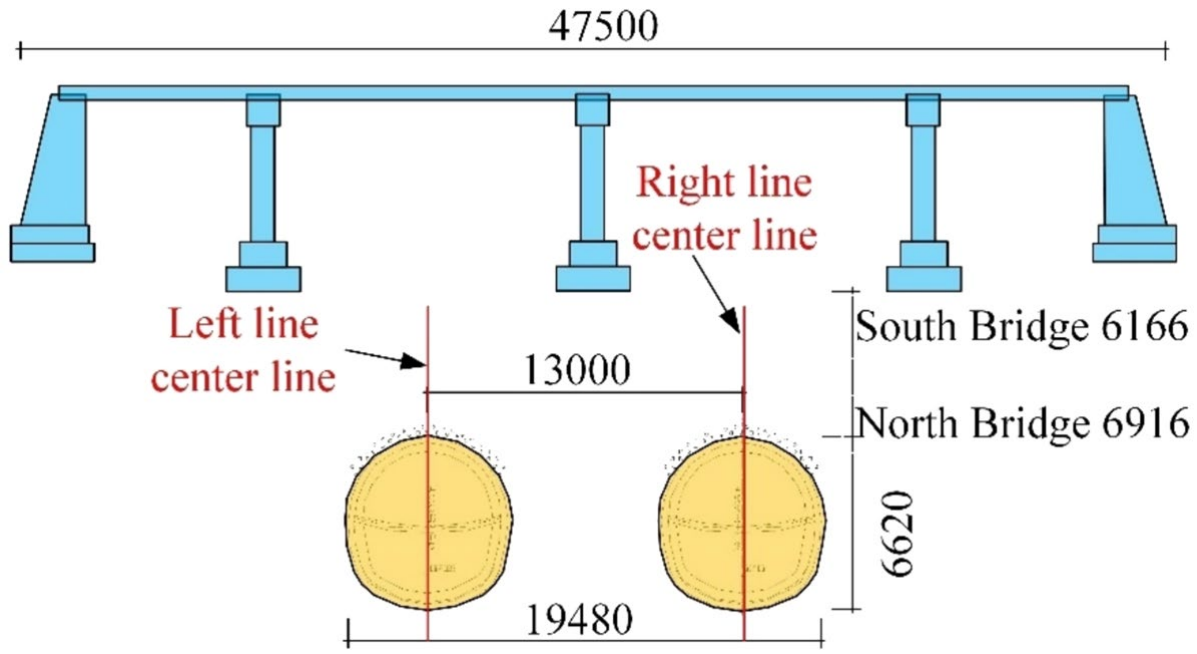
Interval tunnel vertical crossing beijing road turning bridge south and north bridge profile location relationship map.

This area is part of the Urumqi River Basin. Due to urban construction, the original terrain and topography have undergone significant changes. There are no natural runoff channels at the surface, except for irrigation canals in the greenbelt, which result in temporary surface runoff. During the survey, no groundwater was encountered at depths up to 40 m, so groundwater impacts on the project can be disregarded. The tunnel crown depth ranges from 8.3 m to 10.4 m, and the primary geological formation through which the tunnel passes consist of pebble layers. The surrounding rock quality is rated as Grade V. The spacing between the left and right lines of the interval tunnel is 13 m. The plan of the section crossing beneath the railway and highway bridges can be found in Fig. 3, with the cross-section details provided in Fig. 4 and Fig. 5.

### Data acquisition

Surveying technicians set up monitoring points at the road surface, rainwater pipelines, and railway bridges to monitor surface settlement, underground pipeline settlement, and the settlement of buildings and structures. For monitoring purposes, a high-precision Trimble DINI03 electronic leveling instrument was used, and field measurements were conducted according to the procedures outlined in the guiding documents. During the observation process, settlement values for each deformation monitoring point were recorded. For analysis, we selected monitoring data from typical settlement points beneath the railway and highway bridges, specifically the data from monitoring points the JCJ-01–01, JCJ-01–02, and JCJ-01–03, to serve as training samples. Data was collected daily from June 18, 2015, to September 15, 2015, resulting in a total of 93 data sets. To save space, only 50 sets of data are provided here, with the data from JCJ-01–02 as an example. The data is shown in Table 1. The maximum value of the measured data is 0.04 mm, and the minimum value is −2.89 mm, which represents the maximum settlement value. Since this dataset consists of initial data and has not yet reached the conditions necessary for settlement prediction, subsequent steps will include data preprocessing, such as data cleaning (handling outliers and data reorganization) and data splitting (into training and testing sets).

**Table 1:** JCJ-01–02 Initial data.

Period	Measured value	Period	Measured value	Period	Measured value
1	−0.72	18	−0.63	35	−0.44
2	−0.82	19	−0.58	36	−0.57
3	−0.75	20	−0.5	37	−0.44
4	−0.89	21	−0.66	38	−0.53
5	−0.68	22	−0.58	39	−0.44
6	−0.48	23	−0.55	40	−0.8
7	−0.66	24	−0.75	41	−0.61
8	−0.5	25	−0.74	42	−0.76
9	−0.78	26	−0.67	43	−0.5
10	−0.56	27	−0.66	44	−0.8
11	−0.69	28	−0.53	45	−0.7
12	−0.69	29	−0.6	46	−0.9
13	−0.71	30	−0.73	47	−0.5
14	−0.69	31	−0.75	48	−0.7
15	−0.56	32	−0.62	49	−0.6
16	−0.48	33	−0.44	50	−0.92
17	−0.58	34	−0.62		

**Fig. 6:**
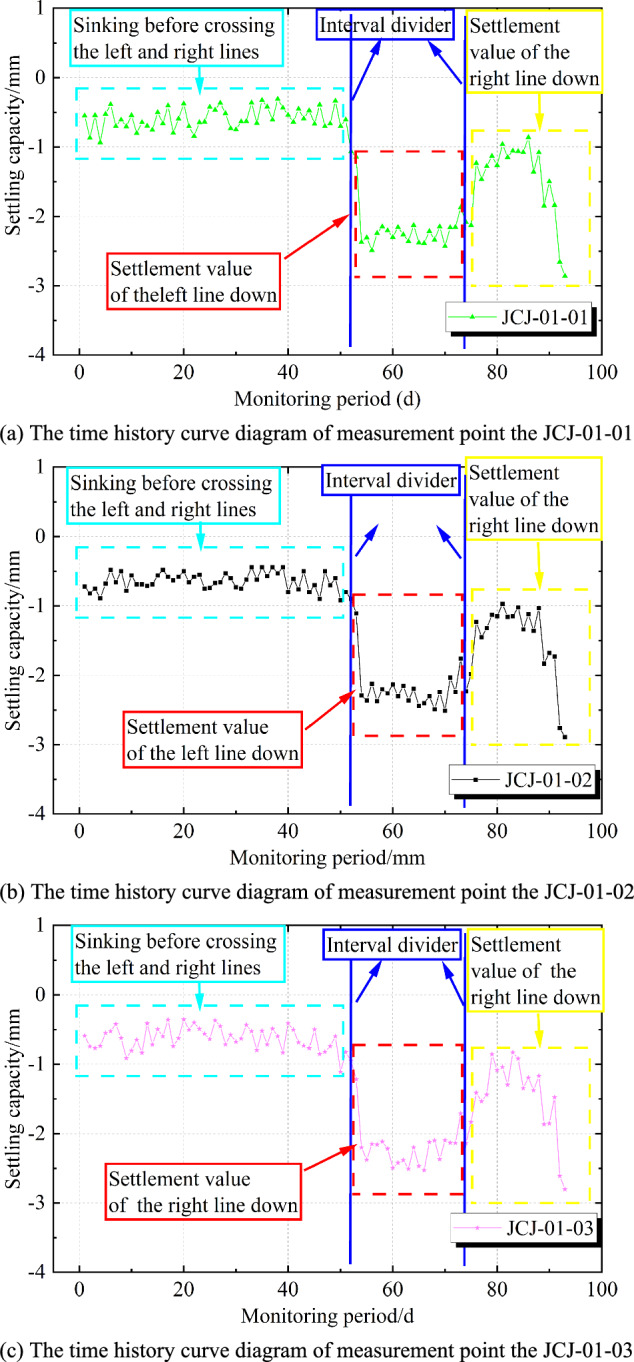

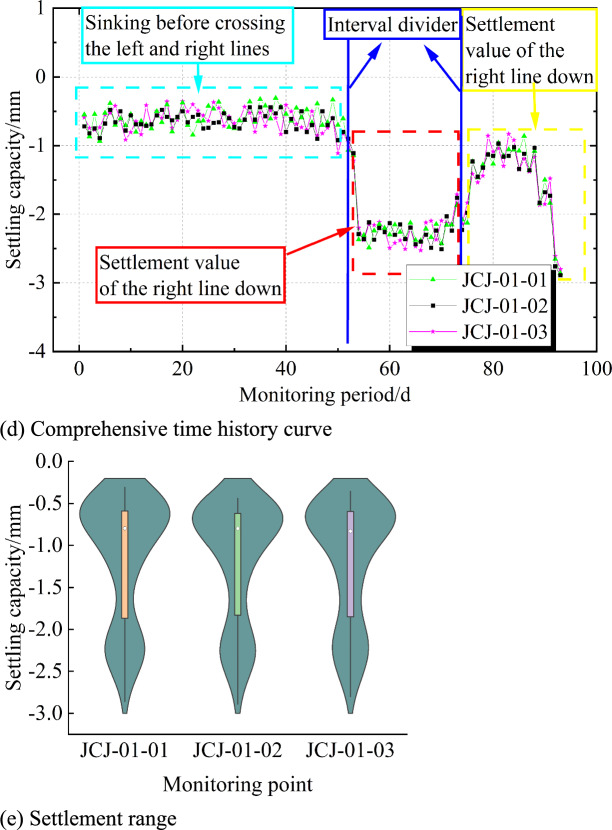
Typical measurement point settlement time history curve and settlement range.

**Fig. 7:**
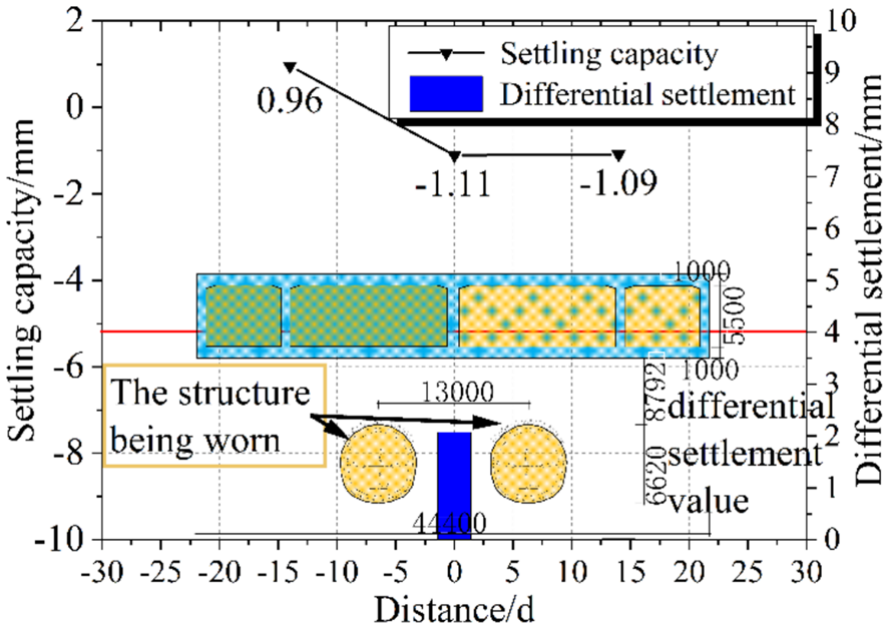
Differential settlement diagram of measurement points for interval tunnel vertical undercrossing railway bridge.

The analysis of Fig 6 and Fig. 7 indicates that the settlement of the bridge structures at the Lu Caogou Bridge and the Beijing Road turning bridge (south and north bridges) under the approach section is relatively small, with the maximum settlement being −2.89 mm and the maximum differential settlement being 2.07 mm. Fig 6 shows the settlement data from June 18, 2015, to September 15, 2015, for each monitoring point, with the following observations: (1) In Fig 6d, the settlement range of the three monitoring points is generally the same. (2) In Fig 6d, the settlement trends of the three monitoring points are overall similar, and the settlement data at different monitoring points exhibit a certain spatial correlation. (3) Among the three monitoring points: On August 10, the left line of the tunnel passing through this location caused a noticeable settlement; After the left line of the tunnel passed through this location on August 31, grouting behind the initial support caused a slight upward movement at the point, after which the settlement stabilized; On August 27, the right line of the tunnel passing through this location caused additional settlement, but the total settlement was relatively small. Therefore, the settlement curve shows significant nonlinearity and fluctuation.

The total settlement under the tunnel passing beneath the high-risk source, Lu Caogou Bridge, is less than -3 mm, with the maximum differential settlement being 2 mm. During the tunnel construction, temporary arches and double-row small guide pipes were added for advanced reinforcement inside the tunnel. No significant cavities or loose zones were observed, and the overall risk was considered controllable. To better illustrate the detailed procedures of data acquisition, Table 2 summarizes the key information. This study collected data from three monitoring points (JCJ-01–01, JCJ-01–02, and JCJ-01–03), systematically demonstrating the spatiotemporal characteristics of the dataset and establishing a reliable foundation for subsequent analysis.

**Table 2:** Summary of dataset information.

Attribute	Description
Measurement point position	Pass under railway and highway Bridges
Data points	3
Settlement point	JCJ-01–01, JCJ-01–02, JCJ-01–03
Monitoring cycle	Once a day
Total number of issues	93
Maximum settlement	−2.89

### Data processing

(1) Wavelet denoising processing.

Before performing formal data prediction, preliminary data processing is required. In wavelet basis functions, there are two options for thresholding: soft thresholding and hard thresholding. This section will explain how to perform the necessary data denoising on the raw data. In the field of signal processing, denoising methods are crucial for improving the quality and accuracy of the signal. This study primarily uses wavelet denoising, which, based on its time–frequency characteristics and multi-resolution features, can effectively process non-stationary signals. To validate the effectiveness of wavelet denoising and compare it with other denoising methods, the study also applies Fast Fourier Transform (FFT) denoising and compares the denoising results of both methods on the experimental dataset.

① Wavelet denoising.

**Fig. 8:**
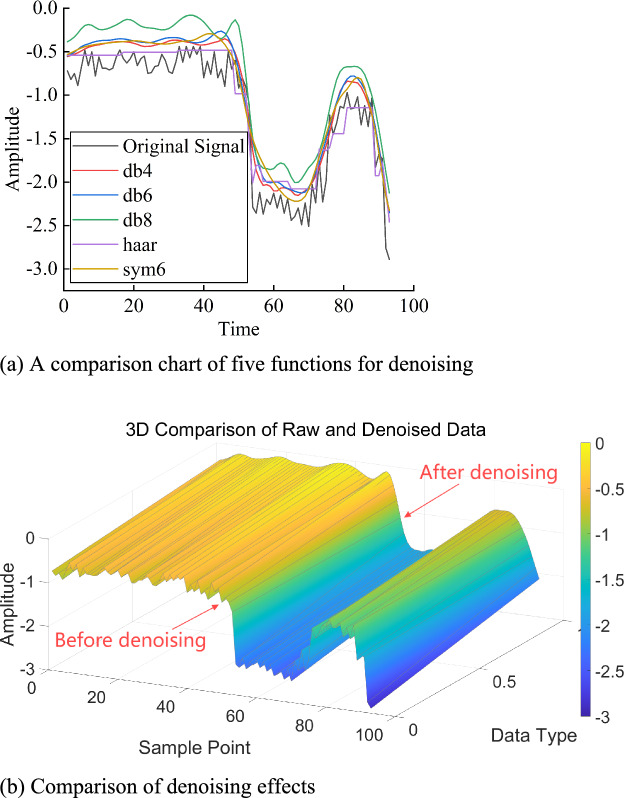
Wavelet denoising.

In wavelet basis functions, there are two choices for thresholding: soft thresholding and hard thresholding, each with different adjustment methods. First, wavelet thresholding denoising is applied to reduce the impact of random factors on the data. This study selects typical wavelets such as db4, db6, db8, haar, and sym6 for 4-level decomposition (with haar wavelet equivalent to db1 wavelet), applying soft thresholding to the high-frequency coefficients. Based on the comparison of the resulting data and graphs, the most suitable db4 wavelet function is chosen for predictive analysis on the processed data, as shown in Fig. 8.

In the denoising comparison chart of Fig. 8a, the curves are presented in the following order: the original measured data curve, the wavelet-denoised data curve, and the denoised data curve with corresponding data points labeled. From the figure, it can be observed that the original data has poor stability and is not a relatively smooth curve. After wavelet denoising, the curve becomes much smoother than the original, and the denoised data generally aligns better with the original measured data. The development trend of the monitoring data is more visually apparent. This indicates that the method effectively removes outlier errors and efficiently extracts the information from the true data. As shown in Fig. 8b, the denoising effect comparison graph, the front part represents the original data, and the rear part shows the denoised data, which forms a smooth curve and visually demonstrates the denoising process. The post-denoising evaluation indicators are shown in Table 3.

To quantitatively evaluate the denoising effect, Signal-to-Noise Ratio (SNR) and Root Mean Square Error (RMSE) are introduced, which serve as important metrics for assessing the effectiveness of signal denoising. According to the data in Table 3, the root-mean-square errors (RMSE) for db4, db6, db8, haar (db1), and sym6 in the denoising results are 0.41417, 0.43874, 0.64881, 0.36482, and 0.48067, respectively. The signal-to-noise ratio (SNR) values are 10.9724, 10.4718, 7.0736, 12.0743, and 9.6791, respectively.

**Table 3:** Evaluation of indicators after denoising using five different functions.

Indicators	db4	db6	db8	haar(db1)	sym6
Threshold	0.92575	1.015	1.2239	0.72767	1.1296
RMSE	0.41417	0.43874	0.64881	0.36482	0.48067
SNR	10.9724	10.4718	7.0736	12.0743	9.6791

As shown in Fig. 8 and Table 3, the comparison of denoising results before and after processing using five wavelet functions reveals the following performance ranking: haar(db1) > db4 > db6 > sym6 > db8. Among them, the waveforms of db4, db6, and db8 are similar and align well with the development trend of the original data, effectively removing anomalous data. For the denoising results under the haar(db1) function, although the denoised error metrics are smaller and the SNR is larger compared to those of other functions, the waveform in the figure exhibits step-like jumps. This is because the haar wavelet is simple and computationally efficient but has poor smoothness and temporal discontinuity, ultimately leading to distortion during signal reconstruction and denoising. Despite haar showing the best results in metrics, the graphical results are practically inferior to those of the db functions. The sym wavelet performs well in denoising stationary signals. For the denoising results under the sym6 function, Fig. 8 shows that the data drop between samples 50–55 is not well-represented, and the data between samples 55–68, which should have been relatively stable, are instead affected by the previous drop and become less stable. In these two segments, the denoised waveform fails to closely follow the variation trend of the original data, and the performance metrics are also suboptimal. In summary, the db4 function is more suitable for wavelet denoising of this dataset.

② FFT denoising.

Compared to wavelet denoising, Fast Fourier Transform (FFT) is a commonly used method for transforming signals from the time domain to the frequency domain. FFT, through spectral analysis of the signal, can identify and remove high-frequency noise in the frequency domain. During the denoising process, FFT sets the high-frequency components of the signal to zero and then recovers the denoised signal back to the time domain using the inverse Fourier transform (IFFT). FFT is more suitable for processing stationary signals, as it is less effective at handling non-stationary signals due to its inability to provide time–frequency localized information. Therefore, FFT denoising is better suited for signals where the noise is primarily concentrated in the high-frequency components.

**Fig. 9:**
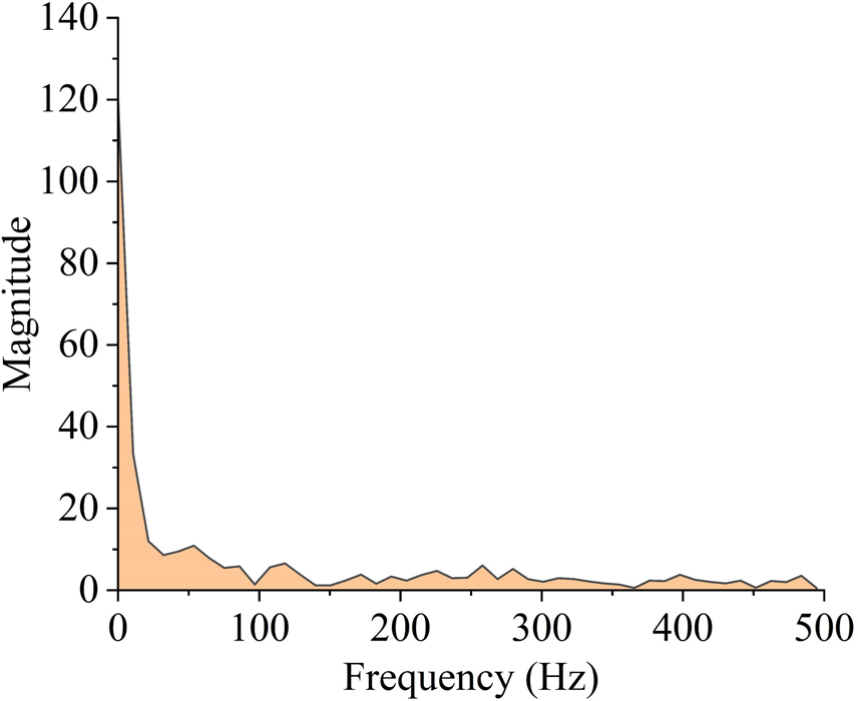
Spectrum plot of FFT.

The sampling frequency is set to 1000 Hz. The figure shows the frequency spectrum obtained by applying Fast Fourier Transform (FFT) to convert the original data from the time domain to the frequency domain. As can be seen from Fig. 9, the effective signal data is primarily concentrated in the low-frequency range of 0–100 Hz, while the noise typically manifests as high-frequency components. Therefore, it is necessary to preserve the low-frequency components and remove the high-frequency components for denoising. Therefore, by setting an appropriate cutoff frequency and removing components above this frequency, the cutoff frequencies are configured as 50, 100, 150, and 200 based on Fig. 9.

**Fig. 10:**
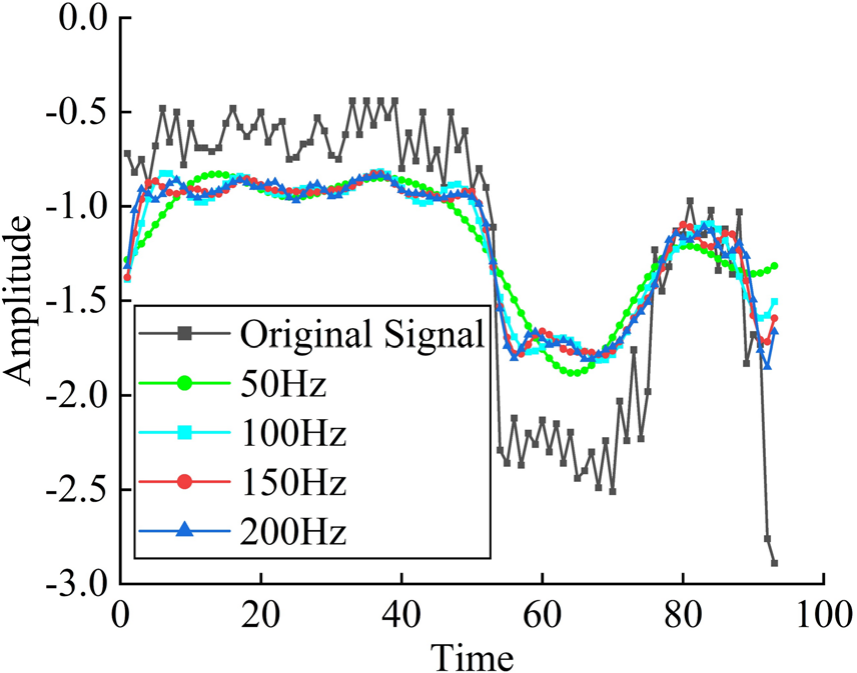
Comparison of FFT denoising.

Figure 10 presents a comparison of the data before and after denoising using Fast Fourier Transform (FFT). It can be observed that the denoising result using FFT is not entirely ideal. The denoised data becomes relatively smoother, reducing the impact of noise. Between 0–50 Hz and 55–70 Hz, the data before and after denoising exhibit the same trend, but with some phase shift. After the "large-scale drop" at the 50 th data point, the data between 55–70 Hz shows the same trend before and after denoising, but again with a phase shift, and the same is observed for the data between 76–90 Hz. It can be concluded that FFT is more suitable for denoising stationary signals, as it can effectively replicate the trend of the original data. However, in cases with "large-scale drops," the denoised result inevitably introduces phase shifts. This issue could be improved by adjusting the algorithm, but it also confirms that FFT denoising is better suited for processing stationary signals.

Table 4 presents the root-mean-square error (RMSE) and signal-to-noise ratio (SNR) at four cutoff frequencies: 50, 100, 150, and 200. Wavelet denoising and Fast Fourier Transform (FFT) exhibit similar performance in terms of RMSE; however, in terms of SNR, FFT is significantly lower than wavelet denoising. This indicates that wavelet denoising still outperforms FFT in terms of performance metrics. Therefore, based on these results, this study will use the wavelet denoising outcomes as the foundational data for subsequent data prediction.

**Table 4:** Parameter settings and post-denoising metrics of FFT.

Sampling Frequency (Hz)	Cutoff Frequency (Hz)	RMSE (mm)	SNR
1000	50	0.45106	4.0126
	100	0.41306	4.777
	150	0.40127	5.0286
	200	0.39356	5.197

(2) Phase Space Reconstruction.

**Fig. 11:**
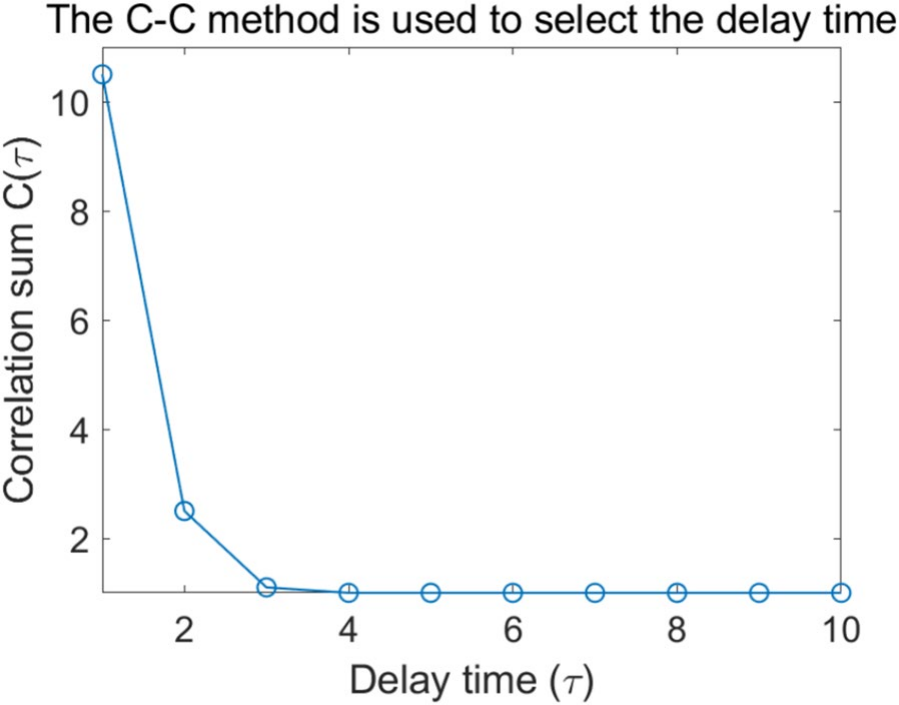
Determining the delay time.

Since the monitoring data is a one-dimensional time series, phase space reconstruction is required on the denoised data. The delay time is determined using the C–C method, as shown in Fig. 11. The first local minimum determines the optimal delay time of 4. Because the relationships between time series data exhibit short-term dependencies, smaller delay times are better at capturing the dynamic features. However, larger delay times cause the embedding space to become sparse, which reduces the model’s prediction accuracy. Therefore, to better capture the detailed trends of data changes in the model learning process, the final delay time is determined to be 1.

**Fig. 12:**
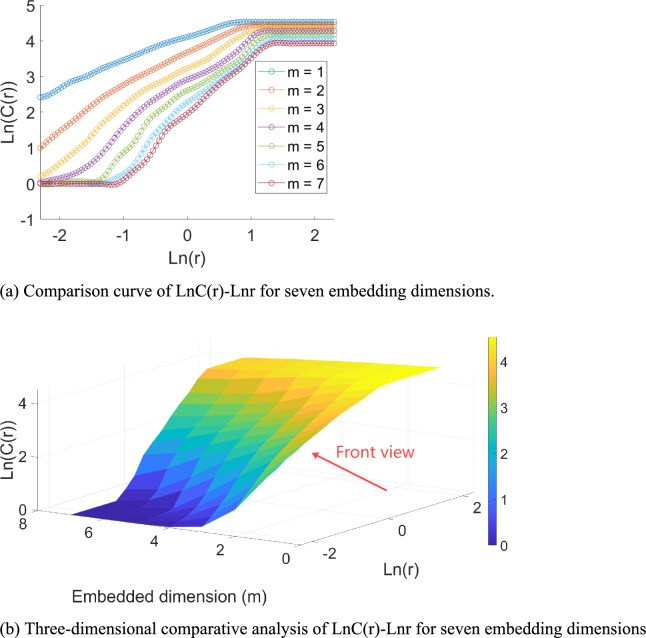
LnC(r)-Lnr curve.

The GP algorithm calculates the saturated correlation dimension to confirm the chaotic behavior of the data. The embedding dimension *m* takes values from 1 to 7, and the LnC(r)-Lnr relationship curve is shown in Fig. 12.

**Fig. 13:**
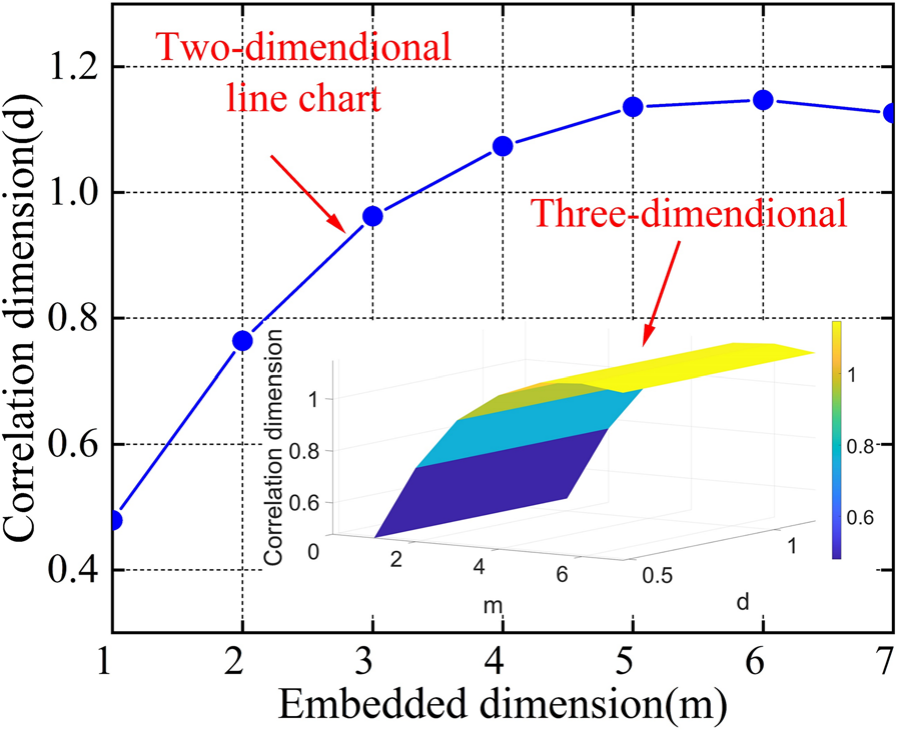
Relationship between correlation dimension and embedding dimension.

A segment of the LnC(r)-Lnr relationship curve in Fig. 12, which approximates a straight line, is selected for the scale-free interval Lnr $\in$[−0.5,1.5]. The slope of the fitted line represents the correlation dimension, and the relationship between correlation and embedding dimensions is shown in Fig. 13. As the embedding dimension grows, the correlation dimension saturates at 1.13626, confirming the chaotic behavior of the time series. The optimal embedding dimension is finally determined to be m = 5. To save space, only 30 sets of reconstructed data are provided. Each column forms a state vector in the phase space, with the first four columns serving as the feature input values for phase space reconstruction, labeled as Feature 1, Feature 2, Feature 3, and Feature 4. The fifth column represents the output value, which corresponds to the data of the fifth day, predicted based on the monitoring data from the previous four days, reflecting the evolutionary result of the state vector. By constructing the five-dimensional embedding space, the time dependency of the system’s state is preserved, while the phase space expansion reveals the nonlinear interactions between state variables, providing a feature space that maintains the topological structure for subsequent nonlinear prediction in machine learning. The data is shown in Table 5.

**Table 5:** The JCJ-01–02 reconstructed data of PSR.

Feature 1	Feature 2	Feature 3	Feature 4	Output
−0.385	−0.3617	−0.3299	−0.2861	−0.2349
−0.3617	−0.3299	−0.2861	−0.2349	−0.198
−0.3299	−0.2861	−0.2349	−0.198	−0.1858
−0.2861	−0.2349	−0.198	−0.1858	−0.1954
−0.2349	−0.198	−0.1858	−0.1954	−0.221
−0.198	−0.1858	−0.1954	−0.221	−0.2429
−0.1858	−0.1954	−0.221	−0.2429	−0.2465
−0.1954	−0.221	−0.2429	−0.2465	−0.24
−0.221	−0.2429	−0.2465	−0.24	−0.2273
−0.2429	−0.2465	−0.24	−0.2273	−0.2089
−0.2465	−0.24	−0.2273	−0.2089	−0.1906
−0.24	−0.2273	−0.2089	−0.1906	−0.1678
−0.2273	−0.2089	−0.1906	−0.1678	−0.1417
−0.2089	−0.1906	−0.1678	−0.1417	−0.1273
−0.1906	−0.1678	−0.1417	−0.1273	−0.1309
−0.1678	−0.1417	−0.1273	−0.1309	−0.1496
−0.1417	−0.1273	−0.1309	−0.1496	−0.179
−0.1273	−0.1309	−0.1496	−0.179	−0.2052
−0.1309	−0.1496	−0.179	−0.2052	−0.2181
−0.1496	−0.179	−0.2052	−0.2181	−0.2244
−0.179	−0.2052	−0.2181	−0.2244	−0.2281
−0.2052	−0.2181	−0.2244	−0.2281	−0.2326
−0.2181	−0.2244	−0.2281	−0.2326	−0.2421
−0.2244	−0.2281	−0.2326	−0.2421	−0.2486
−0.2281	−0.2326	−0.2421	−0.2486	−0.2459
−0.2326	−0.2421	−0.2486	−0.2459	−0.2328
−0.2421	−0.2486	−0.2459	−0.2328	−0.2072
−0.2486	−0.2459	−0.2328	−0.2072	−0.1736
−0.2459	−0.2328	−0.2072	−0.1736	−0.1389
−0.2328	−0.2072	−0.1736	−0.1389	−0.1089

In summary, this section completes the wavelet denoising and phase-space reconstruction of the original data. As shown in Table 6, the specific processing steps and results are as follows: ①Wavelet denoising: Based on the 4-layer decomposition of the db4 wavelet basis function, redundant noise was effectively removed through the soft threshold function, and the rationality of wavelet denoising was verified according to RMSE and SNR; ②Phase-space reconstruction: The C–C method determined the delay time $\tau$ as 1, the GP algorithm determined the embedding dimension m as 5, and a five-dimensional phase-space matrix was constructed. Through these preliminary data preprocessing operations, the authenticity and dynamic characteristics of the prediction data were significantly improved, providing sufficient data dynamic characteristics for subsequent nonlinear prediction.

**Table 6:** Data preprocessing.

Pretreatment steps	Technical details	Effect verification
WTD	1. The db4 function was used for 4-level decomposition	RMSE: 0.41417 SNR: 10.9724
	2. Adopt the soft-threshold strategy	
	3. Remove the noise according to the obtained threshold	
PSR	1. The C–C method determines the time delay	$\tau$: 1 *m*: 5 Inputs: columns 1–4; Output: column 5
	2. The GP algorithm determines the embedding dimension	
	3. Data reconstruction	

### Support vector regression model training

The traditional SVR model and the GSM-SVR model are used for prediction, with a comparison and analysis of their outcomes. The analysis centers on prediction errors and the model’s fit quality, which serve as criteria for assessing its performance. Taking the monitoring data from the 89 th period as an example, the experiment is divided into training and testing sets in a 4:1 ratio. The first 80% of the data is used as the training set, while the remaining data is used as the testing set^57^. Based on the data sequence reconstructed from phase space, the input layer has 4 units, and the output layer has 1 unit for simulation and prediction. In the subsequent steps, PSO-SVR, WOA-SVR, GWO-SVR, MPA-SVR, and LSTM models will be used for further comparison and prediction.

(1) SVR Method Prediction.

**Fig. 14:**
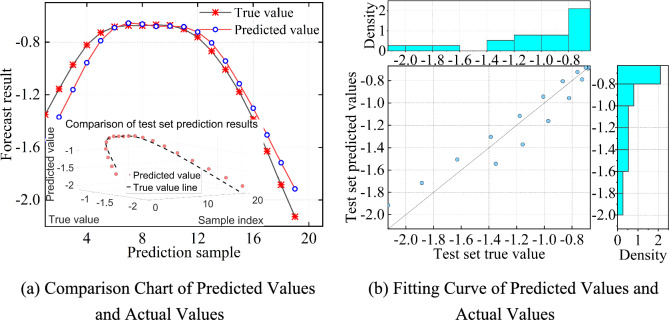
SVR settlement prediction vs. actual values comparison and fitting curve.

Using MATLAB programming for computation, the appropriate model parameters are set, and the prediction is carried out using the JCJ-01–02 monitoring point data. The prediction results obtained using the SVR method are shown in Fig. 14.

(2) GSM-SVR Method Prediction.

**Fig. 15:**
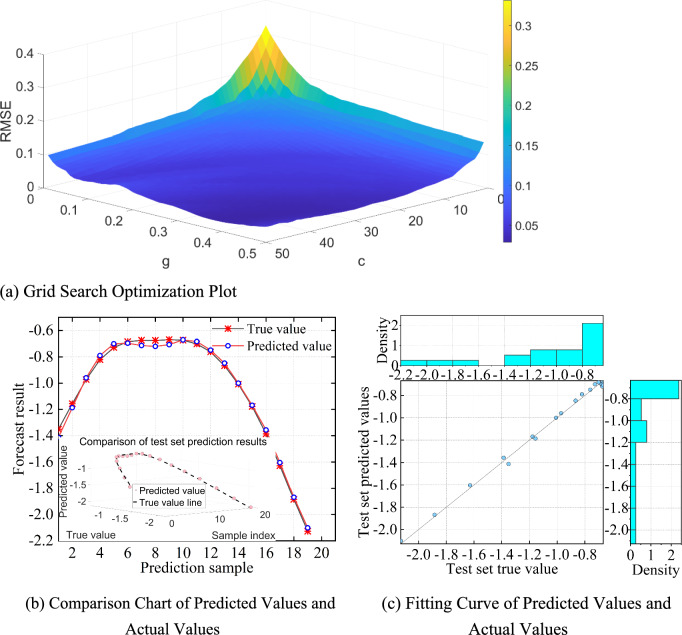
GSM-SVR settlement prediction vs. actual values comparison.

The grid search method can calculate the optimal combination of hyperparameter values and is one of the most widely used SVM parameter optimization techniques^58^. The model depends on the hyperparameters c and g, where a larger c value leads to closer fitting of the training data, potentially causing overfitting. To minimize error, the grid search optimization algorithm (GSM) is used to find the optimal values of c and g. As one of the most common optimization algorithms, c and g are divided into a grid within a specified range, based on a given step size. Then, during the iteration process across all grid points, each selected grid node’s corresponding combinations of c and g are searched, and the model’s MAPE, MAE, RMSE, and goodness-of-fit R^2^ are computed. This yields the best values of c and g, based on optimal cross-validation metrics, avoiding the pitfalls of local optimal solutions and slow convergence that other intelligent algorithms may encounter. GSM-SVR prediction results are shown in Fig. 15.

The GSM method traverses all parameter combinations, optimizing SVR parameters in MATLAB with c and g values ranging from 0.1 to 100, and a maximum of 100 iterations. Shown in Fig. 15a, resulting in the optimal parameters c = 50 and g = 0.38. See Fig. 15b and Fig. 15c for the prediction results. RMSE decreased by 75.14%, showing a significant improvement in fitting compared to the traditional SVR model. The LSTM network, comprising an input, hidden, and output layer, predicts based on the optimized GSM-SVR model.

To validate the superiority of GSM-SVR, other methods will be introduced to verify the accuracy of the GSM-SVR model. These methods include the Particle Swarm Optimization (PSO), Gray Wolf Optimization (GWO), Marine Predators Algorithm (MPA), and Whale Optimization Algorithm (WOA)-optimized SVR models. As shown in Table 7, the parameter settings for the four optimization algorithms are presented, along with key code snippets: The particle swarm size is set to 30, and the hyperparameters c and g are set within the range [0.1, 100], with a maximum number of iterations set to 100. The LSTM network model consists of three parts: the input layer, hidden layer, and output layer, which uses the constructed GSM-SVR model for prediction. The time delay step is set to 5, the prediction step is set to 1, and the network training is conducted using the Adam gradient descent algorithm.

**Table 7:** Optimize the parameter Settings of the algorithm.

%% Optimization algorithm parameter settings	
numPredators = 30;	% Population size setting
maxIter = 100;	% Maximum iteration number
lb = [0.1, 0.1]	% Lower bounds of penalty factor and kernel parameters
ub = [100, 100]	% Upper bounds of penalty factor and kernel parameters
%% Position update and boundary constraints	
for i = 1:numPredators	
% Move randomly towards the optimal solution	
step_size = rand(1,2);	
predators(I, :) = predators(I, :) + step_size.* (bestPredator – predators(I, :));	
% The control parameters are within the search range	
predators(I, :) = max(lb, min(ub, predators(I, :)));	
End	

### Analysis of predictive model adaptability

#### Performance metrics

MAPE, MAE, RMSE, and R^2^ assess and compare the performance of different models. MAPE represents the average deviation from the actual prediction results, MAE is the mean of the absolute errors, and RMSE is used to measure the deviation between the predicted values and the actual values^59^. The expressions for these evaluation metrics are shown in the equations.

23 $$MAPE=\frac{1}{N}\sum_{i=1}^{N}\left|\frac{{y}_{i}^{mea}-{y}_{i}^{pre}}{{y}_{i}^{mea}}\right|\times 100\%$$

24 $$MAE=\frac{1}{N}\sum_{i=1}^{N}\left|{y}_{i}^{mea}-{y}_{i}^{pre}\right|$$

25 $$RMSE=\sqrt{\frac{1}{N}\sum_{i=1}^{N}{\left({y}_{i}^{mea}-{y}_{i}^{pre}\right)}^{2}}$$

26 $${R}^{2}=1-\frac{\sum_{i=1}^{N}{\left({y}_{i}^{mea}-{y}_{i}^{pre}\right)}^{2}}{\sum_{i=1}^{N}{\left({y}_{i}^{mea}-E\left[{y}^{mean}\right]\right)}^{2}}$$

where *y*_*i*_^*mea*^ is the monitored data, *y*_*i*_^*pre*^ is the predicted result, *E*[*y*^*mean*^] is the average value of *y*_*i*_^*mea*^, *N* is the sample size, and *k* is the number of independent variables.

#### Impact of Different Embedding Dimensions on Performance

We selected the range of m from 3 to 8, with a delay time τ = 1, and used MAPE, MAE, RMSE, and R^2^ as evaluation metrics. The experimental dataset consists of 93 data points from the JCJ-01–02 monitoring station. For the embedding dimension, 80% of the dataset is used as the training set, and 20% is used as the testing set.

**Fig. 16:**
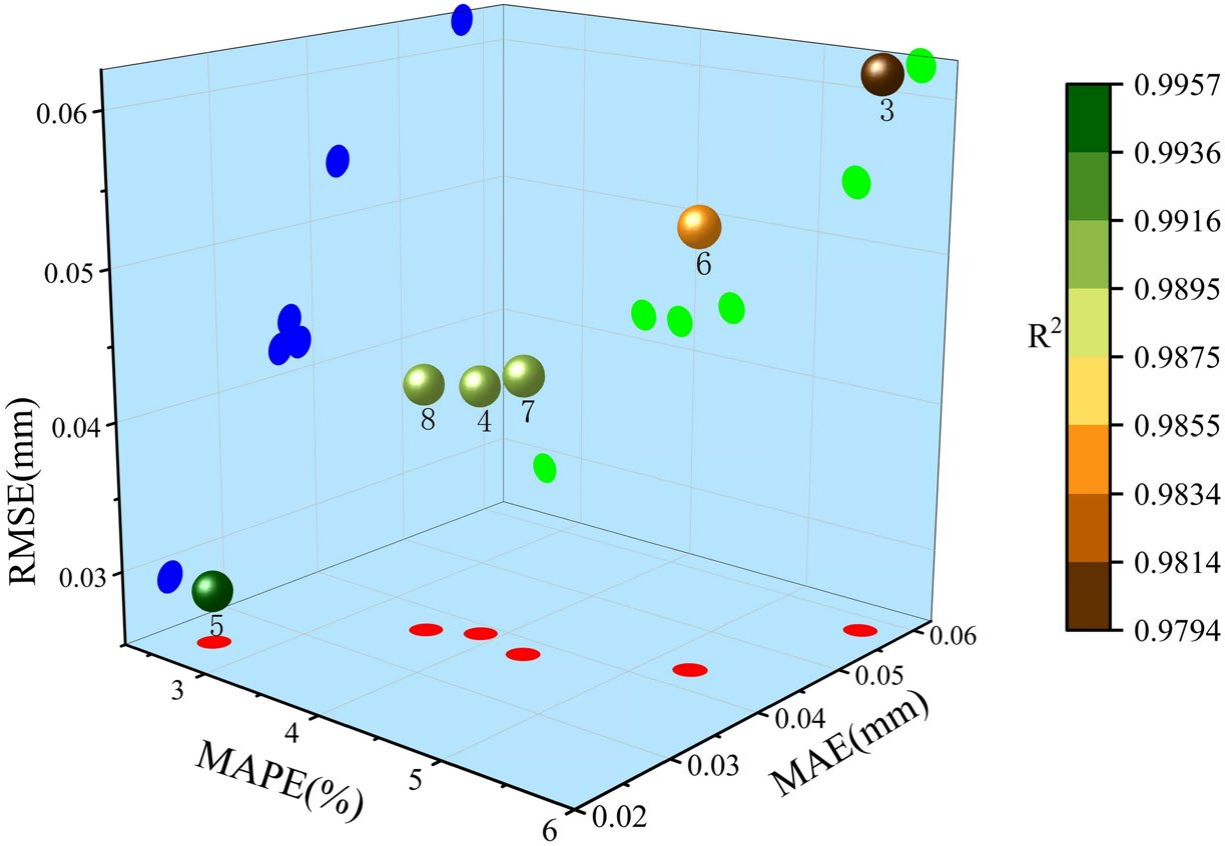
Three-dimensional error plots for six embedding dimensions.

Figure 16 and Table 8 present a visual comparison of errors and performance evaluation metrics for six different embedding dimensions. Figure 16 illustrates the model prediction results under six embedding dimensions, showing performance across three metrics: RMSE, MAPE, and MAE, while the color scale indicates the model’s goodness of fit (R^2^). In the three-dimensional space, each data point represents a prediction result for a specific dimension, with its position determined by the values of MAPE, MAE, and RMSE. Projections on the XY, YZ, and XZ planes correspond to mappings of the three-error metrics, and the color reflects the corresponding goodness of fit, R^2^. The higher the R^2^, the more the color tends toward green, indicating better model fit; the lower the R^2^, the more the color tends toward red, indicating poorer model fit. From the figure, it is evident that the 4 th, 7 th, and 8 th models, located near the center of the cube, exhibit moderate error performance across the six embedding dimensions. In contrast, the 6 th model performs the worst, followed by the 3rd model, both of which show lower R^2^ values and poor fit. The best performance is observed in the 5 th model, corresponding to an embedding dimension of 5, where the model achieves optimal results. Therefore, both the Fig. 16 and Table 8 not only provide direct evidence for selecting the embedding dimension but also offer valuable references for subsequent model optimization and selection.

**Table 8:** Performance evaluation metrics under six embedding dimensions.

Embedding dimensions		MAPE/%	MAE/mm	RMSE/mm	R^2^
m	3	5.733	0.057	0.062	0.979
	4	3.872	0.039	0.042	0.991
	5	2.655	0.025	0.029	0.996
	6	5.284	0.043	0.054	0.984
	7	4.313	0.038	0.044	0.990
	8	3.555	0.036	0.042	0.991

#### Prediction results for JCJ-01–01 monitoring point

**Fig. 17:**
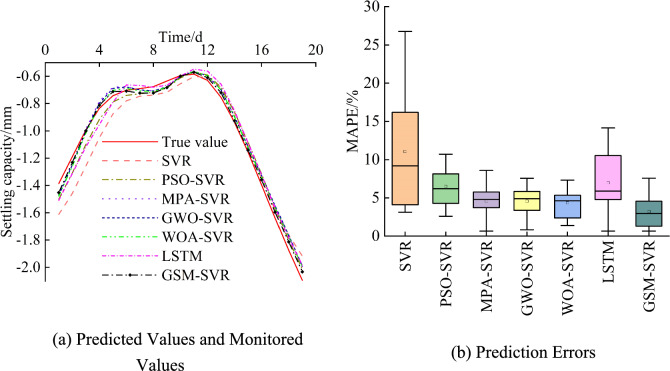
Prediction results for monitoring Point JCJ-01–01.

When predicting the settlement at the JCJ-01–01 monitoring point, the prediction process for the JCJ-01–02 monitoring point was conducted simultaneously. The structure and parameters of each model were the same as those used for the JCJ-01–02 monitoring point case. Figure 17 shows the prediction results and monitored values for each model at the JCJ-01–01 monitoring point. The following observations can be made: (1) In comparison with the six other models, the traditional SVR model produces predictions that are farther from the monitored values, with the largest error and mean error. (2) The predictions from all the optimized models are quite similar, but the MPA-SVR model exhibits a larger fluctuation range in prediction errors. (3) The GSM-SVR model produces predictions that are closest to the monitored values, with the smallest concentration range of errors and the smallest mean error, demonstrating great stability.

Table 9 presents the average prediction performance metrics for each model at the JCJ-01–01 monitoring point. The bold numbers in the table represent the smallest error values within each group. From the table, we can observe the following: (1) The error metrics for the traditional SVR model are the largest. Among the optimized SVR models, the GWO-SVR and WOA-SVR models show relatively smaller performance metrics, while the GSM-SVR model has the smallest error metrics across the board. (2) In comparison with the existing models, the GSM-SVR model achieves a reduction in MAPE by 26.38% to 71%, a reduction in MAE by 30.93% to 70.73%, a reduction in RMSE by 31.02% to 72.28%, and an improvement in R^2^ by 0.78% to 9.25%. This analysis suggests the GSM-SVR model is optimal.

**Table 9:** Performance metrics of each model for monitoring point JCJ-01–01.

Model	Prediction accuracy			
	MAPE/%	MAE/mm	RMSE/mm	R^2^
SVR	11.06	0.109	0.135	0.909
PSO-SVR	6.51	0.065	0.071	0.975
MPA-SVR	4.59	0.049	0.057	0.984
GWO-SVR	4.57	0.049	0.057	0.983
LSTM	6.97	0.071	0.081	0.967
WOA-SVR	4.35	0.046	0.054	0.985
GSM-SVR	3.21	0.032	0.037	0.993

#### Prediction results for JCJ-01–02 monitoring point

**Fig. 18:**
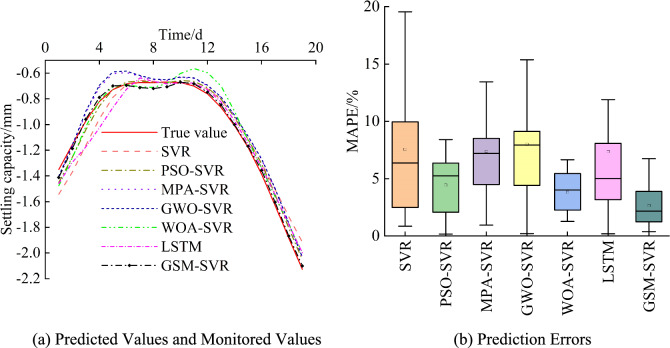
Prediction results for monitoring point JCJ-01–02.

Figure 18 shows the predicted and actual settlement values, along with the prediction errors, for each model at the JCJ-01–02 monitoring point. The following observations can be made: (1) The predicted values from all models closely follow the trend of the actual values, with the predicted values generally aligning with the actual values, indicating that the hyperparameters are reasonably set. (2) The SVR model exhibits larger fluctuations in prediction errors, with the maximum and mean errors being the highest. (3) Among the SVR models, the GWO-optimized model shows a larger fluctuation range in prediction errors. The GSM-SVR model, however, has the smallest maximum and mean prediction errors and is the most stable.

For a more detailed comparison of the forecast performance of the respective models, Table 10 shows the mean prediction performance metrics for each model at the JCJ-01–02 monitoring point. The bold numbers in the table represent the smallest error values within each group. The following conclusions can be inferred: (1) The performance metrics of all prediction models are lower than those of the SVR model, suggesting that the algorithm-optimized SVR models outperform the traditional SVR model. (2) The GSM-SVR model consistently achieves the smallest performance metrics. Compared to the other models, the MAPE value is reduced by 30.29% to 64.83%, the MAE value is reduced by 43.64% to 72.11%, the RMSE value is reduced by 45.66% to 75.14%, and the R^2^ value increases by 1.06% to 7.16%. This proves that GSM-SVR is an optimum model.

**Table 10:** Performance metrics of each model for monitoring point JCJ-01–02.

Model	Prediction accuracy			
	MAPE/%	MAE/mm	RMSE/mm	R^2^
SVR	7.55	0.088	0.115	0.929
PSO-SVR	4.44	0.050	0.055	0.981
MPA-SVR	7.37	0.073	0.081	0.965
GWO-SVR	7.98	0.078	0.086	0.960
LSTM	7.36	0.076	0.097	0.949
WOA-SVR	3.81	0.044	0.051	0.985
GSM-SVR	2.65	0.025	0.029	0.996

#### Prediction results for JCJ-01–03 monitoring point

**Fig. 19:**
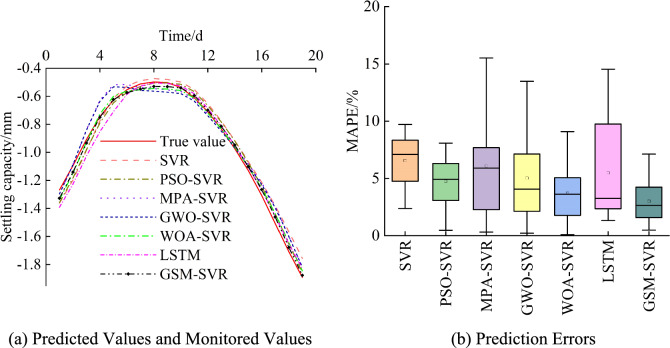
Prediction results for monitoring point JCJ-01–03.

When predicting the settlement at the JCJ-01–03 monitoring point, the prediction process for the JCJ-01–02 monitoring point was conducted simultaneously. The structure and parameters of each model were the same as those used for the JCJ-01–02 monitoring point case. Figure 19 shows the prediction results and monitored values for each model at the JCJ-01–03 monitoring point. The following observations can be made: (1) All models’ predictions generally align with the monitored values, with the GSM-SVR model’s predictions being the closest. (2) The predictions from the optimized SVR models and the LSTM network are generally similar. (3) The traditional SVR model produces the largest mean prediction error. Although the maximum errors from the MPA-SVR and GWO-SVR models are larger than those of the traditional SVR model, their mean errors are smaller. The GSM-SVR model consistently shows the smallest maximum, mean, and minimum prediction errors.

To further analyze the model performance, Table 11 presents the average performance metrics for each model at the JCJ-01–03 monitoring point. The bold numbers in the table represent the smallest error values within each group. From the table, the following observations can be made: (1) The traditional SVR model and MPA-SVR model have relatively larger error metrics, while the GWO-SVR model and LSTM network have similar error metrics. (2) The GSM-SVR model consistently achieves the smallest error metrics. The MAPE value is reduced by 18.76% to 54.17%, the MAE value is reduced by 21.17% to 60.21%, the RMSE value is reduced by 24.54% to 60.54%, and the R^2^ value is improved by 0.4% to 2.93%.

**Table 11:** Performance metrics of each model for monitoring point JCJ-01–03.

Model	Prediction accuracy			
	MAPE/%	MAE/mm	RMSE/mm	R^2^
SVR	6.57	0.066	0.076	0.967
PSO-SVR	4.77	0.045	0.052	0.985
MPA-SVR	6.11	0.052	0.064	0.977
GWO-SVR	5.03	0.045	0.055	0.983
LSTM	5.48	0.051	0.065	0.976
WOA-SVR	3.71	0.033	0.040	0.991
GSM-SVR	**3.01**	**0.026**	**0.030**	0.995

#### Comprehensive prediction results

(1) Comparative analysis of average prediction performance.

To evaluate the overall performance of each model, Table 12 presents the average prediction performance metrics for each model across three monitoring points. The bold numbers in the table represent the smallest error values within each group. The conclusions that can be drawn from the table are as follows: (1) The average error metrics for the traditional SVR model are the largest, indicating that the SVR models optimized by algorithms outperform the traditional SVR model. (2) Among the optimized SVR models, the WOA-SVR and PSO-SVR models achieve relatively smaller average error metrics. (3) The GSM-SVR model consistently produces the smallest average error metrics. The MAPE value, MAE value, and RMSE value are reduced by 25.25% to 64.72%, 32.93% to 68.61%, and 34.43% to 70.53%, respectively. Additionally, the R^2^ value is improved by 0.75% to 6.39%.

**Table 12:** Average performance metrics of each model.

Model	Prediction accuracy			
	MAPE/%	MAE/mm	RMSE/mm	R^2^
SVR	8.39	0.088	0.109	0.935
PSO-SVR	5.24	0.053	0.059	0.980
MPA-SVR	6.02	0.058	0.067	0.975
GWO-SVR	5.86	0.057	0.066	0.975
LSTM	6.60	0.066	0.081	0.964
WOA-SVR	3.96	0.041	0.049	0.987
GSM-SVR	2.96	0.028	0.032	0.995

(2) Paired t-test.

To further validate the comparative performance of the models, this study conducted paired t-tests for all models based on their MAPE values from three monitoring points, using GSM-SVR results as the reference baseline. Table 13 shows the results of the paired t-test. From Table 13 it can be seen that: ①Overall, the mean differences and t-values of the t-test results for all models were less than 0, and all P-values were less than 0.05, indicating that compared with the other six intelligent prediction models, GSM-SVR showed significant superiority; ②The mean difference and t-value of the t-test results for the SVR model were the smallest, indicating that the performance of SVR was the lowest among all models, further confirming the necessity of optimizing the SVR model with GSM; ③Performance comparison of the seven models: GSM-SVR > WOA-SVR > PSO-SVR > GWO-SVR > MPA-SVR > LSTM > SVR, and the paired t-test results were consistent with the prediction results.

**Table 13:** Significance test results.

Comparison model	Mean difference	95% confidence interval	t	p
SVR	−16.30	[−23.15, −9.46]	−5.003	0.000
PSO-SVR	−6.85	[−11.06, −2.63]	−3.409	0.003
MPA-SVR	−9.20	[−13.90, −4.50]	−4.112	0.001
GWO-SVR	−8.71	[−12.99, −4.43]	−4.273	0.000
WOA-SVR	−3.00	[−4.84, −1.15]	−3.414	0.003
LSTM	10.94	[−18.52, −3.37]	−3.304	0.007

#### Comparison of denoised and non-denoised prediction results

**Fig. 20:**
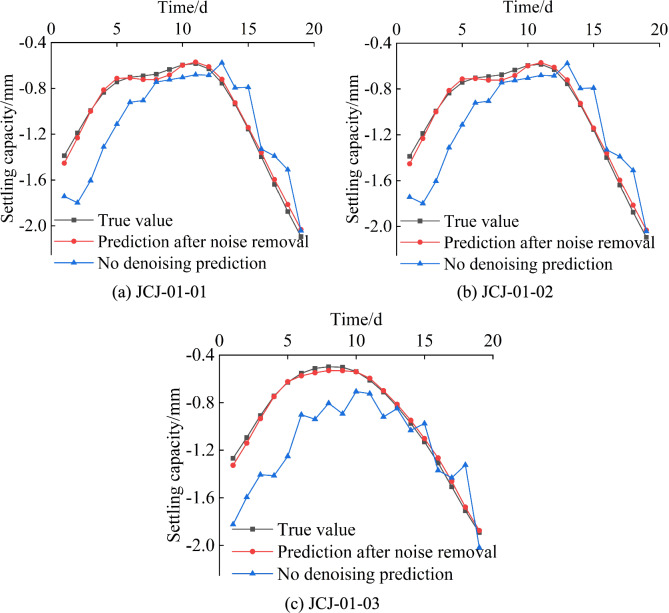
Comparison of prediction results between denoised and non-denoised data.

To validate the effectiveness of wavelet de-noising, the original and denoised data are compared. We applied the GSM-SVR prediction to the raw, unprocessed data without denoising. As illustrated in Fig. 20, although the prediction of the non-denoised data shows a certain development trend similar to the monitoring data, the error is significantly larger compared to the prediction based on the denoised data, and the fit is also lower.

The effect of de-noising on the prediction precision of GSM-SVR is obvious, as shown in Table 14. The results indicate that: (1) MAPE: The MAPE value before denoising is relatively high, especially for the JCJ-01–03 model, which reaches 39.99%. This indicates that the prediction error is a significant proportion of the actual value. After data denoising, the MAPE value significantly decreases, and the MAPE of all models drops below 3%. This suggests that the data denoising process significantly enhanced the model’s accuracy, effectively reducing the relative error between predictions and actual values. (2) MAE: Before denoising, the MAE varies across the three points, with JCJ-01–01 having an MAE of 0.247 mm and JCJ-01–03 having an MAE of 0.300 mm. After de-noising, the MAE is obviously reduced, and the error is near 0.03 mm, which shows that the difference between the prediction and the real value is significantly reduced. (3) RMSE: Before denoising, the RMSE shows relatively high values at all three monitoring points, especially for JCJ-01–03, which reaches 0.361 mm, indicating that the model’s prediction errors are relatively large. After denoising, the RMSE also decreases significantly, approaching 0.03 mm, which shows a substantial improvement in the model’s prediction accuracy. (4) R^2^: Before denoising, the R^2^ value is relatively low, particularly for JCJ-01–02, which is only 0.358, indicating poor data fitting. After denoising, the R^2^ values for all three monitoring points increase significantly, with JCJ-01–02 reaching 0.996. This indicates a substantial improvement in data fitting after denoising, allowing the model to better predict the actual data.

**Table 14:** Average performance metrics of denoised and non-denoised predictions for three measurement points.

Model		Prediction accuracy			
		MAPE/%	MAE/mm	RMSE/mm	R^2^
JCJ-01–01	Non-Denoised	25.68	0.247	0.304	0.466
	Denoised	3.21	0.032	0.037	0.993
JCJ-01–02	Non-Denoised	26.27	0.268	0.315	0.358
	Denoised	2.65	0.026	0.029	0.996
JCJ-01–03	Non-Denoised	39.99	0.300	0.361	0.413
	Denoised	3.01	0.026	0.030	0.995

#### Fitting regression analysis of predicted values and actual values

**Fig. 21:**
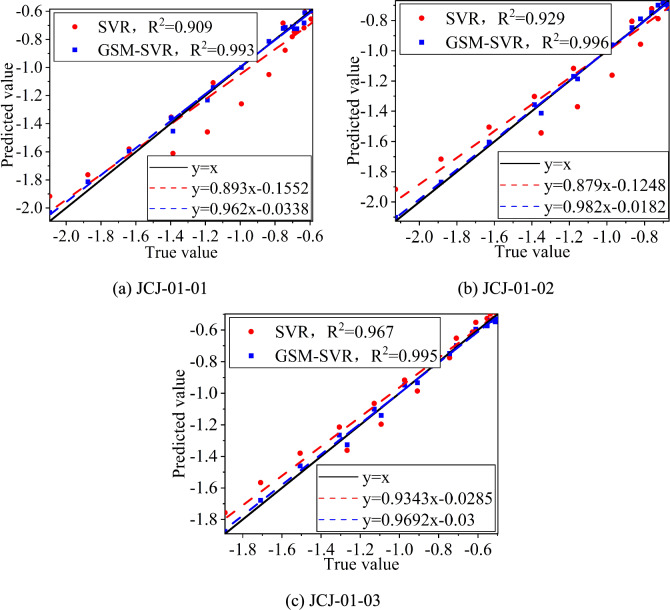
Comparison of fitting between point-SVR and GSM-SVR.

**Fig. 22:**
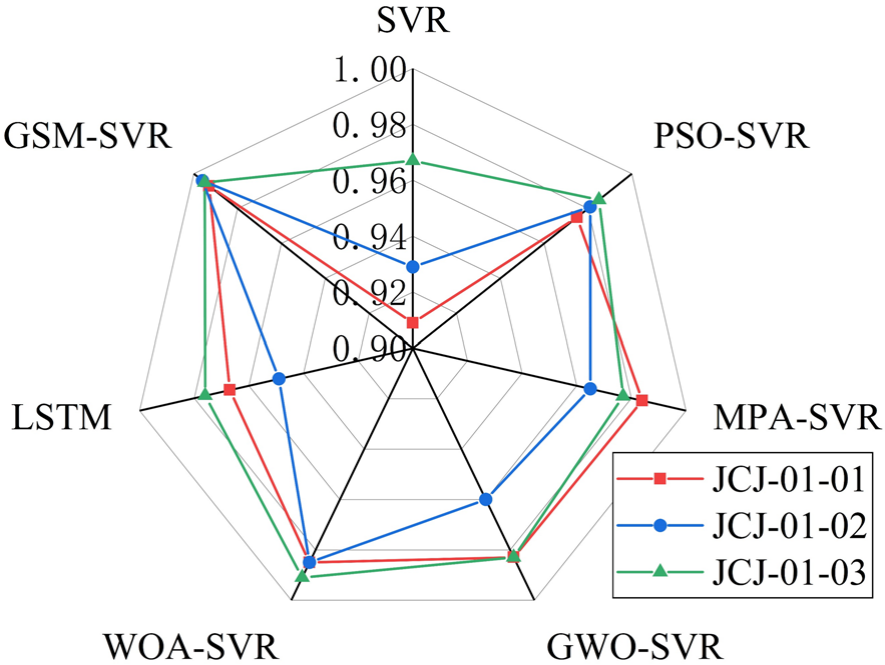
Radar chart of fitting degree evaluation.

To better capture the prediction outcomes of each model, Fig. 21 illustrates the prediction results of the traditional SVR and GSM-SVR models on the same test set. In the fitting plot of the true values and predicted values of the test set, the sample points of the GSM-SVR model are distributed on both sides of the perfect fit line (y = x), and the predicted values are closer to the diagonal compared to those of the traditional SVR model. The satisfactory fitting values (R^2^ = 0.993; R^2^ = 0.996; R^2^ = 0.995) of the three points JCJ-01–01, JCJ-01–02, and JCJ-01–03 further demonstrate that the predictions of the GSM-SVR model are more consistent with the true values. The evaluation metrics for the fitting performance of the aforementioned models are summarized in the radar chart shown in Fig. 22. The results show that GSM-SVR has a higher correlation coefficient than the others, which shows that GSM-SVR model has better prediction performance than the others. That is to say, GSM can improve the forecast performance of conventional SVR. Compared with the other six models, it is also proved that the proposed method is robust in the forecast of settlement.

It is clear from the above discussion that the GSM-SVR model produces the minimum forecast error and the best fit. The reason for this is that the GSM optimization algorithm can explore all possible parameter combinations through cross-validation, with automatic enumeration by the algorithm, ensuring that no possible parameter configurations are overlooked. This makes it superior to conventional optimization algorithms. Furthermore, the key advantage of the grid search method is its simplicity, comprehensiveness, and unbiased nature, making it suitable for cases with a small hyperparameter space or sufficient computational resources. The computational requirements of this study are fully compatible with those of the grid search method. Moreover, the model improves its ability to capture autocorrelation in settlement data during training, allowing it to more effectively identify trends in settlement changes. These analyses all demonstrate that the GSM-SVR model performs best in predicting subway settlement data.

## Conclusion

This study proposes a GSM-SVR-based subway settlement prediction model based on phase space reconstruction, with core innovations including: (1) using wavelet denoising to select the optimal db4 function for data preprocessing, and comparing it with fast Fourier transform denoising, revealing that wavelet denoising achieves better denoising performance; (2) employing phase space reconstruction to extend the one-dimensional time series into a five-dimensional feature space; (3) utilizing grid search method (GSM) to optimize the hyperparameters of the SVR model.

The results indicate that: (1) the average prediction accuracy of all seven models exceeds 93%, validating the effectiveness of model selection in subway settlement prediction; (2) the intelligent optimization models significantly improve the ability to capture nonlinear features compared to the traditional SVR, with WOA-SVR and PSO-SVR showing stable performance, while MPA-SVR exhibits fluctuations due to its global search mechanism being prone to local optima in high-dimensional space; (3) GSM-SVR achieves optimal performance with an average error of 2.96%, improving accuracy by 25.25%–64.72% compared to the other models. Its grid search-based global hyperparameter optimization strategy demonstrates the best stability, confirming the superiority of the enumeration cross-validation method in surface settlement prediction; (4) The phase space reconstruction enhances the dynamic characteristics of the data, effectively improving the ability to capture complex nonlinear settlement features, providing a highly reliable prediction tool for engineering monitoring.

Outlook: (1) The current study does not consider other influencing factors of subway settlement on the prediction of cumulative settlement. In future work, data such as excavation depth and weather conditions, which affect subway settlement, can be incorporated into the model training to improve the prediction accuracy. (2) The model can be further refined. There is still room for improvement in feature engineering and hyperparameter adjustment mechanisms. Future research will focus on the coupling analysis of spatial mechanisms across monitoring points and multidimensional influencing factors to enhance the engineering applicability and physical interpretability of the settlement prediction model.

## Supplementary Information

Supplementary Information.

## Data Availability

The datasets used and/or analysed during the current study are available from the corresponding author on reasonable request.

## Code availability

The implementation of optimization algorithms (PSO, GWO, MPA, and WOA) developed in this research can be obtained by contacting the corresponding author with justified academic requests.
